# A Phase I Clinical Trial with *Ex Vivo* Expanded Recipient Regulatory T cells in Living Donor Kidney Transplants

**DOI:** 10.1038/s41598-018-25574-7

**Published:** 2018-05-09

**Authors:** James M. Mathew, Jessica H.-Voss, Ann LeFever, Iwona Konieczna, Cheryl Stratton, Jie He, Xuemei Huang, Lorenzo Gallon, Anton Skaro, Mohammed Javeed Ansari, Joseph R. Leventhal

**Affiliations:** 1 Department of Surgery, Comprehensive Transplant Center, Feinberg School of Medicine, Northwestern University, Chicago, IL 60611 USA; 2 Department of Microbiology and Immunology, Feinberg School of Medicine, Northwestern University, Chicago, IL 60611 USA; 3 Mathews Center for Cellular Therapy, Northwestern Memorial Hospital, Chicago, IL 60611 USA; 4 Department of Medicine, Division of Nephrology, Feinberg School of Medicine, Northwestern University, Chicago, IL 60611 USA; 5 TRACT Therapeutics, Inc; 125W. Oak Street; Suite D, Chicago, IL 60610 USA

## Abstract

There is considerable interest in therapeutic transfer of regulatory T cells (Tregs) for controlling aberrant immune responses. Initial clinical trials have shown the safety of Tregs in hematopoietic stem cell transplant recipients and subjects with juvenile diabetes. Our hypothesis is that infusion(s) of Tregs may induce transplant tolerance thus avoiding long-term use of toxic immunosuppressive agents that cause increased morbidity/mortality. Towards testing our hypothesis, we conducted a phase I dose escalation safety trial infusing billions of *ex vivo* expanded recipient polyclonal Tregs into living donor kidney transplant recipients. Despite variability in recipient’s renal disease, our expansion protocol produced Tregs which met all release criteria, expressing >98% CD4^+^CD25^+^ with <1% CD8^+^ and CD19^+^ contamination. Our product displayed >80% FOXP3 expression with stable demethylation in the FOXP3 promoter. Functionally, expanded Tregs potently suppressed allogeneic responses and induced the generation of new Tregs in the recipient’s allo-responders *in vitro*. Within recipients, expanded Tregs amplified circulating Treg levels in a sustained manner. Clinically, all doses of Treg therapy tested were safe with no adverse infusion related side effects, infections or rejection events up to two years post-transplant. This study provides the necessary safety data to advance Treg cell therapy to phase II efficacy trials.

## Introduction

Kidney transplantation is the treatment of choice for most causes of end stage renal diseases^1,2^. While transplantation is effective in replacing the non-functional kidney, disparity between donor and recipient major histocompatibility antigens results in massive activation of the recipient’s immune system that, if left unchecked, leads to subsequent rejection of the organ. To prevent this, patients must take immunosuppressive drugs (IS) for life, generally a combination of agents including a calcineurin inhibitor (CNI), and corticosteroids^3–6^. However, dependence on IS tempers the substantial benefit obtained from transplantation^1–13^. Specifically, CNIs are nephrotoxic, a side effect of significant concern in transplantation while steroids exacerbate osteoporosis and hyperlipidemia, and cause avascular osteonecrosis. Development of alternate therapies that help to minimize the need for lifelong immunosuppression, or to eliminate them entirely through the induction of tolerance, are therefore of great interest.

Regulatory CD4^+^CD25^+^ T cells (Treg) derived from the thymus and/or peripheral tissues have been demonstrated to broadly control T cell reactivity^14^. Importantly, Tregs have been shown to control immune responsiveness to alloantigens and contribute to operational tolerance in pre-clinical transplantation models^15–20^. Initial efforts to evaluate the therapeutic effects of Tregs in humans have focused upon stem cell transplant recipients in an effort to control graft versus host disease (GVHD) or to treat autoimmune diseases^21–24^. There have been limited efforts to harness the therapeutic potential of Tregs in clinical solid organ transplantation (SOT), due to challenges with the isolation and expansion of Tregs for clinical use. In this manuscript we have tested the hypothesis whether we can develop a protocol for the isolation and large-scale expansion of human Tregs and conduct a phase I clinical trial of Treg adoptive cell transfer therapy following living donor kidney transplantation. Using our unique expansion protocol and experience with personalized cellular therapies;^25^ we enrolled nine living donor kidney transplant recipients into a three-tiered dose ranging study (0.5 × 10^9^, 1.0 × 10^9^, 5 × 10^9^ Tregs / recipient). We subsequently monitored these patients post infusion and found maintained levels of Tregs throughout the study period. Importantly, there were no serious adverse events associated with Treg infusion and no clinical rejection events up to two years post-transplant. In addition, we report an in depth characterization of the nine Treg products infused into each recipient.

## Results

### Subjects

**Figure 1:**
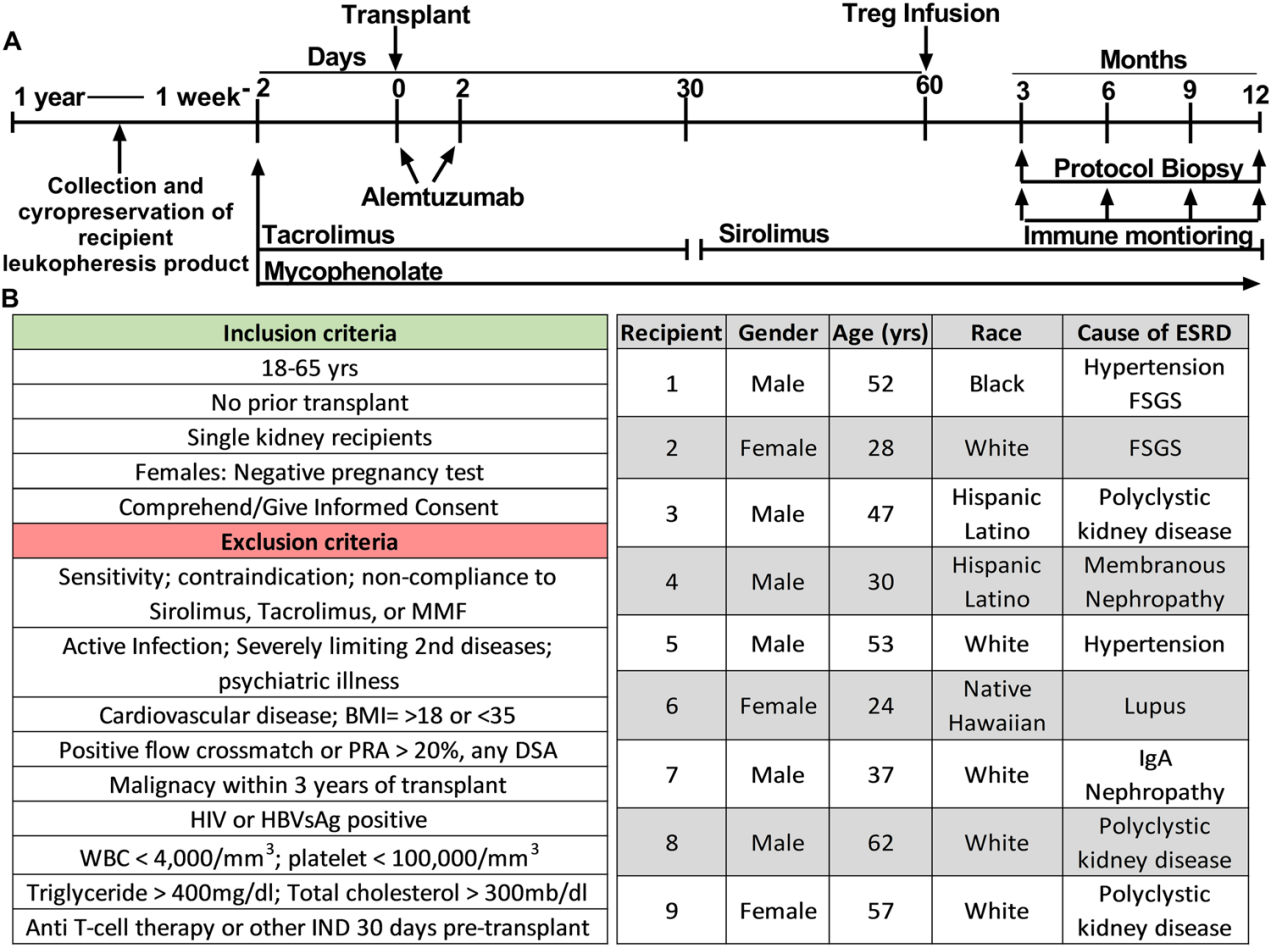
Clinical Protocol and subjects in the Phase I safety trial: (**A**) Outline of the clinical trial design from initiation to one-year follow-up. Although not listed patients were given intravenous corticosteroids of 500 mg pre-operatively, and weaned to 250 mg, 125 mg and 0 mg on post-operative days 1, 2 and 3 respectively. (**B**) The inclusion and exclusion criteria. The target population was adult recipients of living donor renal allografts that would not require hemodialysis during the first week following renal transplantation. (**C**) Demographics of living donor kidney transplant recipients receiving expanded autologous Tregs. Number of Tregs received by the Subjects: 1–3: 0.5 × 10^9^, 4–6: 1 × 10^9^, and 7–9: 5 × 10^9^.

In late 2014, we initiated a Phase 1 trial of autologous polyclonal expanded TReg Adoptive Cell Therapy (TRACT) in living donor kidney transplant recipients, recruited through a Northwestern University Institutional Review Board (IRB) and FDA approved protocol after obtaining informed consent (NCT 02145325, IND 15898). This was a nonrandomized dose-ranging study with 3 tiers of cell dosing (0.5, 1, and 5 × 10^9^ cells infused/recipient) with 3 recipients per dosing tier. The clinical protocol for the trial is graphically shown in (Fig. 1A). The inclusion and exclusion criteria considered in recipient recruitment are described in (Fig. 1B). The target population was adult recipients of living donor renal allografts that would not need hemodialysis during the first week following renal transplantation. Ten subjects were enrolled into this study. One subject was withdrawn following enrollment due to the development of medical problems that precluded him as a kidney transplant candidate. The nine recipients displayed diversity in sex, ethnicity, and cause of end stage renal disease (Fig. 1C). Recipients underwent leukopheresis, approximately one month prior to the kidney transplant. This leukopheresis product was cryopreserved for later isolation and manufacturing of Tregs. Recipients received alemtuzumab induction at the time of kidney transplantation to achieve lymphodepletion, based on our pre-clinical observation that a reduction in circulating effector T cells worked synergistically with Treg infusion to prolong allograft survival^15^. During the immediate post-transplant period, the recipients were placed on a tacrolimus and mycophenolate-based immunosuppressive drug therapy for rejection prophylaxis. At two months post-transplant, recipients were fully converted off of tacrolimus and onto the mTOR inhibitor sirolimus with the said conversion completed prior to Treg infusion; calcineurin inhibitors such as tacrolimus are known to block the production of IL-2, a critical survival cytokine for Tregs, and the Treg promoting features of sirolimus have been well described^26–30^. Conversion to sirolimus was successful in all but one patient who developed thrombocytopenia, a known potential side effect of sirolimus. An historical cohort of kidney transplant recipients who received identical induction and maintenance immunosuppression but no Treg infusion was utilized as a control group for comparison of serial immunophenotypic reconstitution over a 12 month period as presented below.

### *Ex Vivo* GMP expansion of recipient Tregs

**Figure 2:**
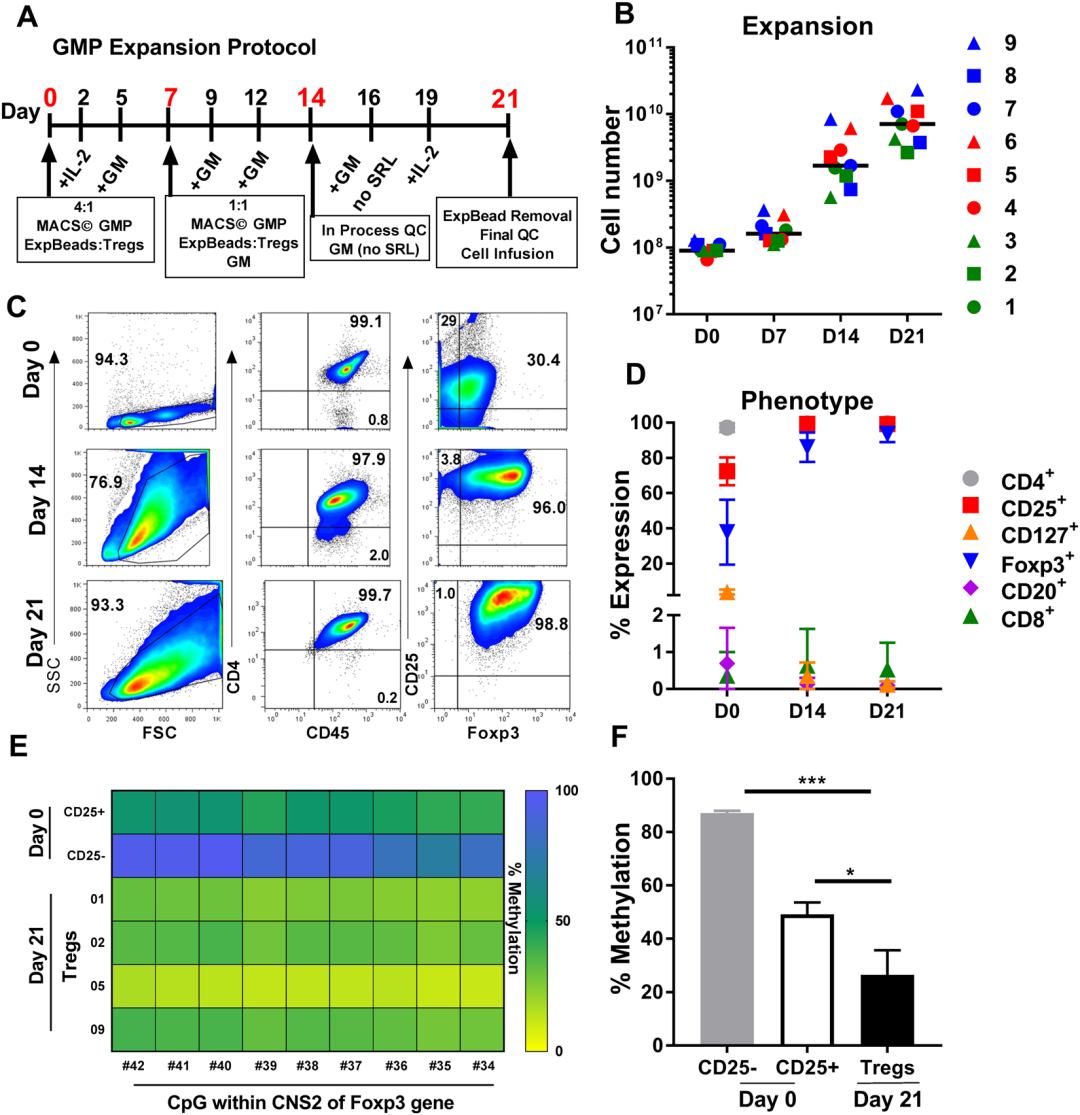
Expansion and Profile of nine expanded Treg products: (**A**) Outline of clinical good manufacturing expansion protocol established at the Mathews Center for Cellular Therapy, Northwestern Memorial Hospital. (**B**) Absolute cell number of all nine Treg products throughout expansion protocol; black bar represents grand median of all products (n=9). (**C**) Representative clinical phenotyping of Treg products, specifically CD4^+^CD25^+^FOXP3^+^ expression on post CD25 enrichment (day 0) as well as days 14 and 21 of culture. (**D**) Represents the average (±SD) of expression of Treg markers CD4; CD25; and FOXP3; also non-Treg markers CD8, CD20, and CD127 throughout expansion protocol (n=9). (**E**) Heat map of clinical Treg products and controls (day 0) depicting the percentage of methylation within 9 CpG sites of the conserved non-coding sequence 2 of FOXP3 gene in the expanded Tregs (TRK- 01–09; n=4). (F) Average (+SD) of the percent methylation in day 0 control products and day 21 expanded Treg products (n=4). ***p < 0.005, *p < 0.05; GM  =  growth medium (described under Materials).

Building upon our previous preclinical work^15^, we optimized a protocol for polyclonal human Treg expansion and transitioned this technology to our GMP facility for large scale clinical manufacturing. The protocol for Treg expansion and quality testing are shown in (Fig. 2A); CD4^+^CD25^+^ Tregs were isolated from patient’s cryopreserved leukopheresis products using all CliniMACS^®^ system and reagents. Treg expansion was initiated with stimulation using MACS GMP Exp-ACT Beads^®^ and IL-2, TGFβ, and Sirolimus on days 0 and 7. Sirolimus was not added to the culture after day 9. In-process evaluation of the cultures was performed on day 14 and final testing was completed on day 21. The cellular product was harvested on day 21 and expansion beads were removed before Treg infusion into recipients (Fig. 2A). We observed robust expansion and met cell dose requirements in all nine Treg manufacturing runs, despite varying causes of end stage renal disease (Fig. 2B).

Our expansion protocol generated Tregs with a classic CD4^+^CD25^+^CD127^−^FOXP3^+^ phenotype with < 1% contaminating CD8^+^ or CD19^+^ cells throughout culture (Fig. 2C and D). The expanded Tregs demonstrated high FOXP3 expression (Fig. 2D) and DNA demethylation of the FOXP3 promoter (Fig. 2E and F), suggesting the expanded cell product retained regulatory properties.

### Expansion alters Treg surface receptor expression

In order for cellular therapies to be effective and survive *in vivo* they must home to sites of inflammation and secondary lymphoid tissues^31–34^ and undergo TCR engagement. Since the total cell product was infused into recipients (~ 98–99% CD4^+^), we characterized the expression of key chemokine and Treg associated receptors on total CD4^+^ T cells (Fig. S1A). Interestingly, we found a significant increase in CXCR4 expression post expansion (Fig. S1B). There was also a trend towards increased CXCR3 and CCR7 expression; however they did not reach statistical significance (Fig. S1B). The Treg-associated markers, CD62L, CTLA4, and GARP were all significantly increased post expansion (Fig. S1C). Finally, we observed a maintained memory phenotype with > 80% cells being CD45RO^+^ and < 10% being CD45RA^+^ post-expansion. Mean fluorescent intensity (MFI) was in accordance with percentages described above (Fig. S2). Taken together, these data suggested that our expanded Tregs had the ability to home to lymph nodes and exert cell contact mediated suppression through CTLA4 engagement.

### Expanded Tregs retained clonal diversity

To assay the breadth and depth of TCR diversity in the expanded Tregs we performed high-throughput sequencing of 6 recipient apheresis samples (before Treg isolation) and day 21 Treg products. We found over 80 percent of the unique V, D, and J rearrangements to be productive, meaning they would produce a functional protein receptor (Fig. S3A). Of those productive rearrangements, we found the clonal diversity, % productive frequency, and top 10-clone frequency significantly decreased post expansion (Fig. S3B). This was likely due to non-expansion of lower frequency clones present in the apheresis product. There was also an increased entropy (i.e. Shannon’s entropy where higher value samples represent greater diversity and lower value samples will share more nucleotide sequences) in the expanded Treg product; however this did not reach statistical significance (Fig. S3B). Overall, Treg receptor diversity was maintained post expansion.

### Expanded Tregs were potently suppressive and induced infectious tolerance

**Figure 3:**
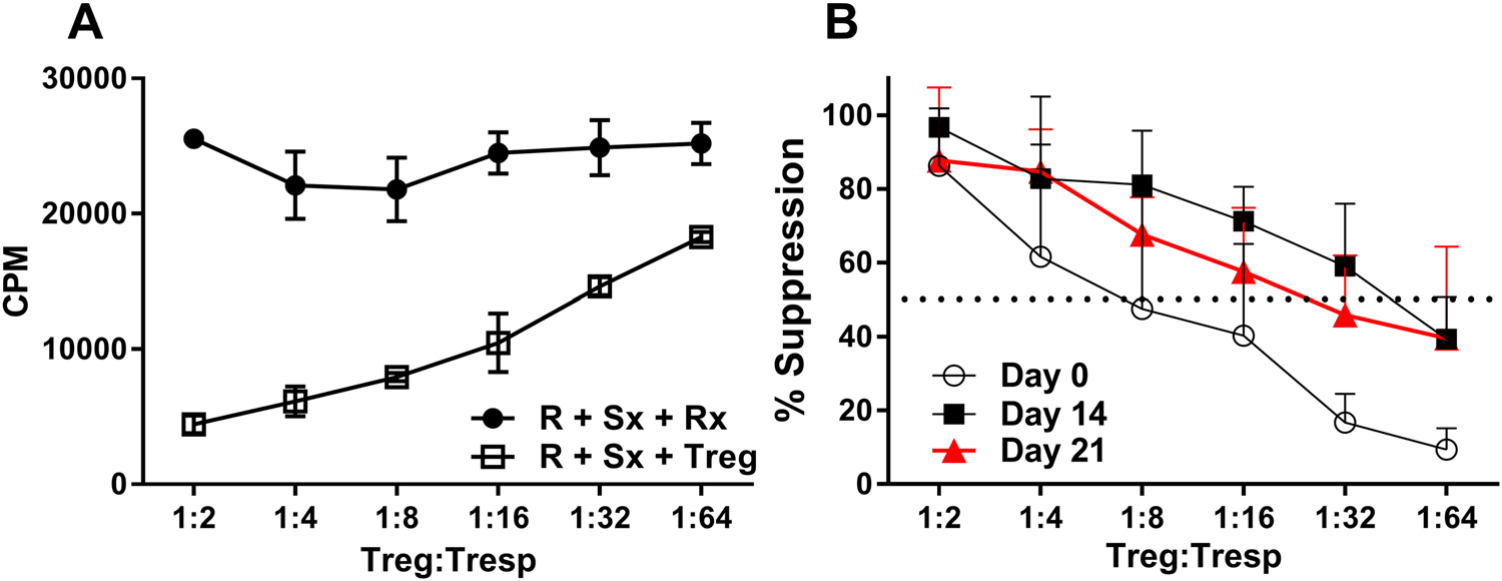
In process testing of Tregs suppressive function: Suppression assays were performed. (R) denotes recipient PBMC’s; (Sx) denotes irradiated healthy allogeneic PBMC’s (stimulators); (Rx) denotes irradiated responder cells used as a control to keep cell number consistent across groups. (**A**) Graph shows raw count per minutes (±SD in triplicate cultures) of thymidine incorporation assay after six days of culture (representative). (**B**) Average (±SD) of percent suppression (n = 9) at varying Treg: Tresp ratios throughout clinical expansion protocol. Percent suppression calculated as described in materials and methods.

We analyzed the suppressive function of our Treg products throughout expansion, on days 0, 14 and 21 using a classic mixed lymphocyte reaction (MLR) suppression assay. Briefly, recipient PBMC’s (R), autologous to the expanded Tregs, were stimulated for 7 days with healthy volunteer allogeneic irradiated PBMCs (Sx) in the presence or absence of expanded Tregs. Treg expansion was associated with an enhanced suppressive potency; when the dose for 50% suppression was assessed, expanded Tregs required 8 fold lower cell number as compared to day 0 Tregs (Fig. 3A and B). MLR inhibition results were comparable on day 14 and day 21 of Treg culture. Therefore, day 14 suppression result was considered the in-process functional test before release and infusion into recipients.

One mechanism by which Tregs mediate suppression is through de novo Treg generation from recipient’s native T cells, a process known as infectious tolerance^35,36^. To assess this *in vitro*, we used the Treg-MLR assay developed in the laboratory^37^. Briefly, suppression assays as described above were performed however, (R) PBMC’s were labeled with CFSE and (Sx) PBMCs with PKH-26 in presence of Tregs that were also labeled with PKH-26. Seven days later the percentage of CD4^+^CD127^−^CD25^+^FOXP3^+^ cells derived from the CFSE^+^PKH-26^negative^ recipient PBMCs that proliferated (diluted CFSE) was calculated (Fig. S4A). We observed a significant increase in the percentage of CD4^+^CD127^−^CD25^+^FOXP3^+^ Tregs generated in the recipient proliferating PBMC when our Treg product was present compared to baseline (no Tregs present) (Fig. S4B).

### Expanded Tregs met all release criteria

As per FDA requirements, prior to release of the expanded Tregs for recipient infusion, cells underwent extensive quality and safety testing (Table 1). It was found that the final formulation met and actually exceeded (Table 1) all the FDA approved release criteria of negative aerobic, anaerobic and fungal contaminations, negative mycoplasma and negative gram stain; <5.0EU/kg endotoxin; >70% viable; >70% CD4^+^ CD25^+^; <10% CD8^+^ and CD19^+^; <3000 Exp-Act® beads/10E8 cells (Table 1). With an eye toward future clinical trials, which require product potency determination prior to release, we included our MLR suppression assay as a part of the release criteria testing. Initial definition of this parameter was loose with a cut off of 50% suppression at a 1:2 ratio, however, we constantly exceeded this parameter obtaining 50% suppression at >1:16 ratio, thus enabling us to further define this potency level for future clinical trials.

**Table 1:** Product specifications of the released expanded Treg products (n = 9)^**^.

Test	Method	Observed Results	Time Testingv Is Performed	Results available prior to release
Bacteriologic sterility	BacTec BD Aerobic and anaerobic system	All Negative	Days 14 & 21	No*
Gram Stain	Mirco Lab SOP	All Negative	Days 14 & 21	Yes
Mycoplasma detection	E-Myco^TM^ PCR	All Negative	Days 14 & 21	Yes
Endotoxin detection	EndoSafe	<0.016EU /kg	Days 14 & 21	Yes
Viability	7AAD staining Flow	>98.3%	Days 14 & 21	Yes
Expression of Treg markers	CD4^+^CD25^+^ Flow cytometry	>99.0% CD4^+^CD25^+^	Days 14 & 21	Yes
FOXP3 Expression	Intracellular Flow cytometry	>80%	Day 21	Yes
Contamination of CD8^+^CD19^+^	CD8^+^ CD19^+^ Flow Cytometry	<0.56% CD8^+^ <0.07% CD20^+^	Days 14 & 21	Yes
Residual Exp-Act Beads	Miltenyi^TM^ Bead Count Assay	<196.33 beads per 1×10^8^ cells	Day 21	Yes
Potency	MLR suppression assay	>87.6% suppression @ (1:2)	Days 14 & 21	No*

*Day 14 in-process testing was taken into consideration for the release of the Treg product.

**All products met or exceeded IND approved release criteria. Prior to unit infusion, the above release criteria were confirmed and reported to the clinician and regulatory agencies.

### Immune monitoring of recipients post Treg infusion

**Figure 4:**
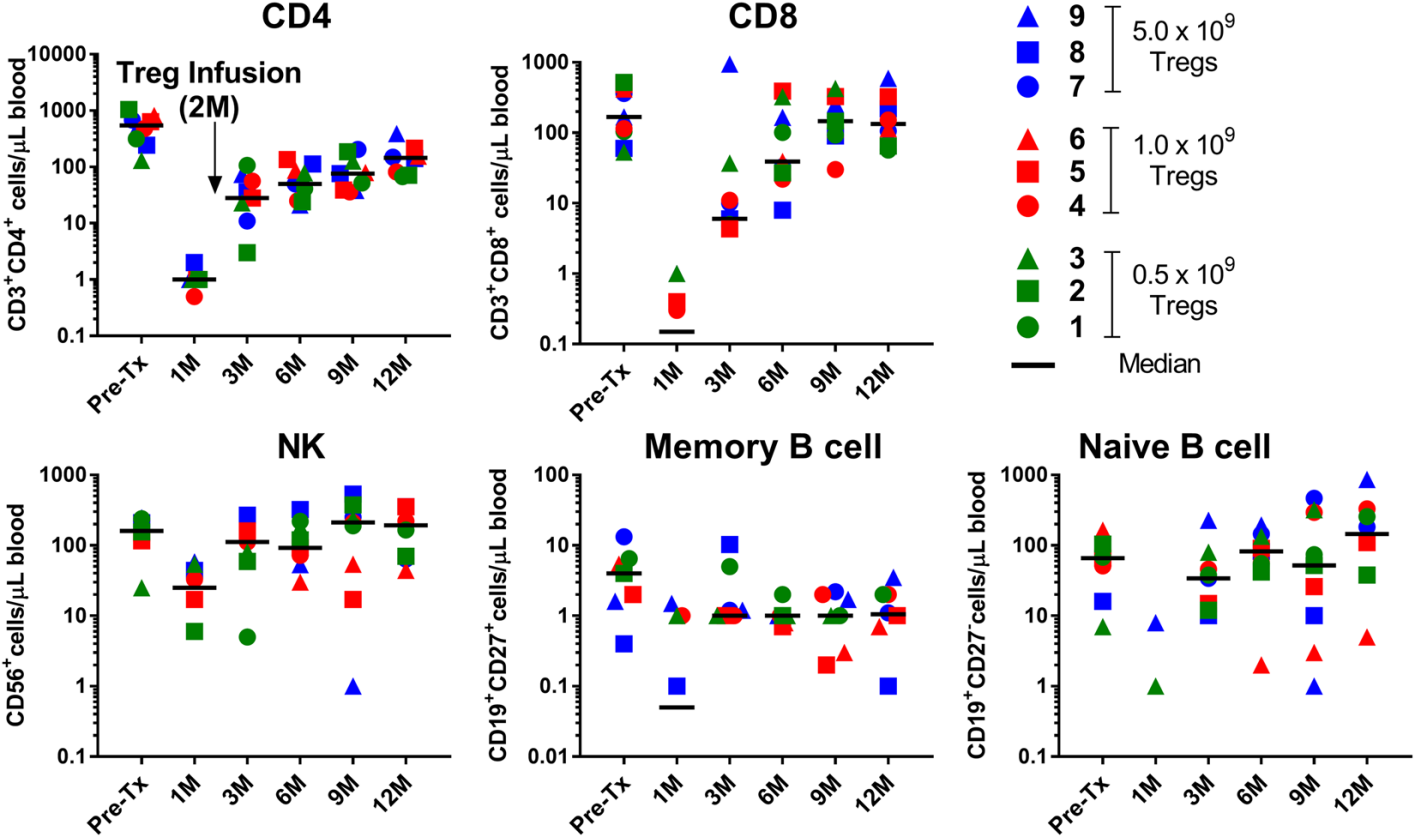
Immune monitoring of recipients post Alemtuzumab induction and Treg infusion. Absolute cell numbers (symbols) and grand median (- line) of T, B, and NK, Tregs per uL of blood from pre-transplant to 12 months post-transplant in all nine recipients (n = 9). Note: Alemtuzumab was given on day 0 and 2 of transplant, and Tregs were infused at two months post-transplant.

**Figure 5:**
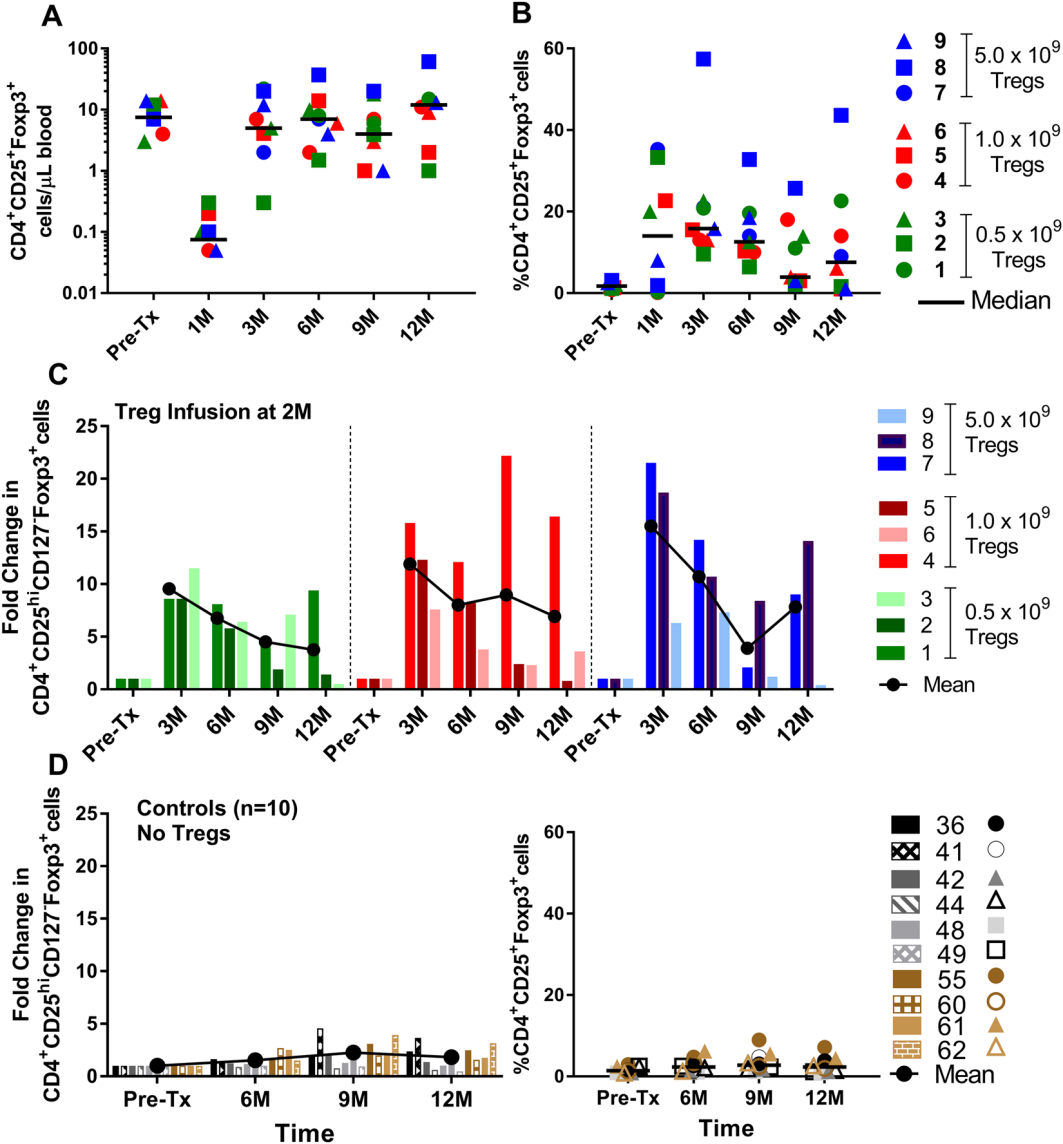
Immune monitoring for Tregs in recipients post Alemtuzumab induction and Treg infusion. (**A**) Absolute cell numbers per uL of blood, and (**B**) percentage of Tregs from pre-transplant to 12 months post-transplant in all nine recipients (n = 9). (**C**) Fold Change in the percentage of Tregs within all nine recipients post Treg infusion when normalized against the pre-transplant percentages. (**D**) The controls were 10 renal transplant recipients treated with the same conditioning and immunosuppression but did not receive Tregs.

As previously mentioned, lymphocyte depletion, specifically the debulking of circulating effector T cells, was deemed important for the efficacy of Treg cell therapy. Therefore, kidney transplant recipients received alemtuzumab induction and thus displayed a significant decrease in all monitored cell subsets including B cells, NK cells and CD14^+^ monocytes at one month post-transplant (Fig. 4). By day +90 there was recovery of NK cells, naïve B cells, (Fig. 4) and CD14^+^ monocytes (not shown). Whereas total CD4^+^ T cells and total CD8^+^ T cells were still significantly reduced at day +90, absolute numbers and percentages of Tregs (Fig. 5A and B, respectively) approached or even exceeded pre-transplant circulating levels, a likely consequence of Treg infusion, which was performed on day +60 post-transplant. Most importantly, Treg infusion resulted in 5–20 fold increase in the percentages of Tregs in all subjects and this was stable in most patients until the end of the follow-up period of one year (Fig. 5C). This was in contrast to minimal enhancement of Tregs in a cohort of historical controls that underwent the exact same lymphodepletion and conversion from CNI’s to sirolimus but did not receive a Treg infusion (n = 10) (Fig. 5D).

**Figure 6:**
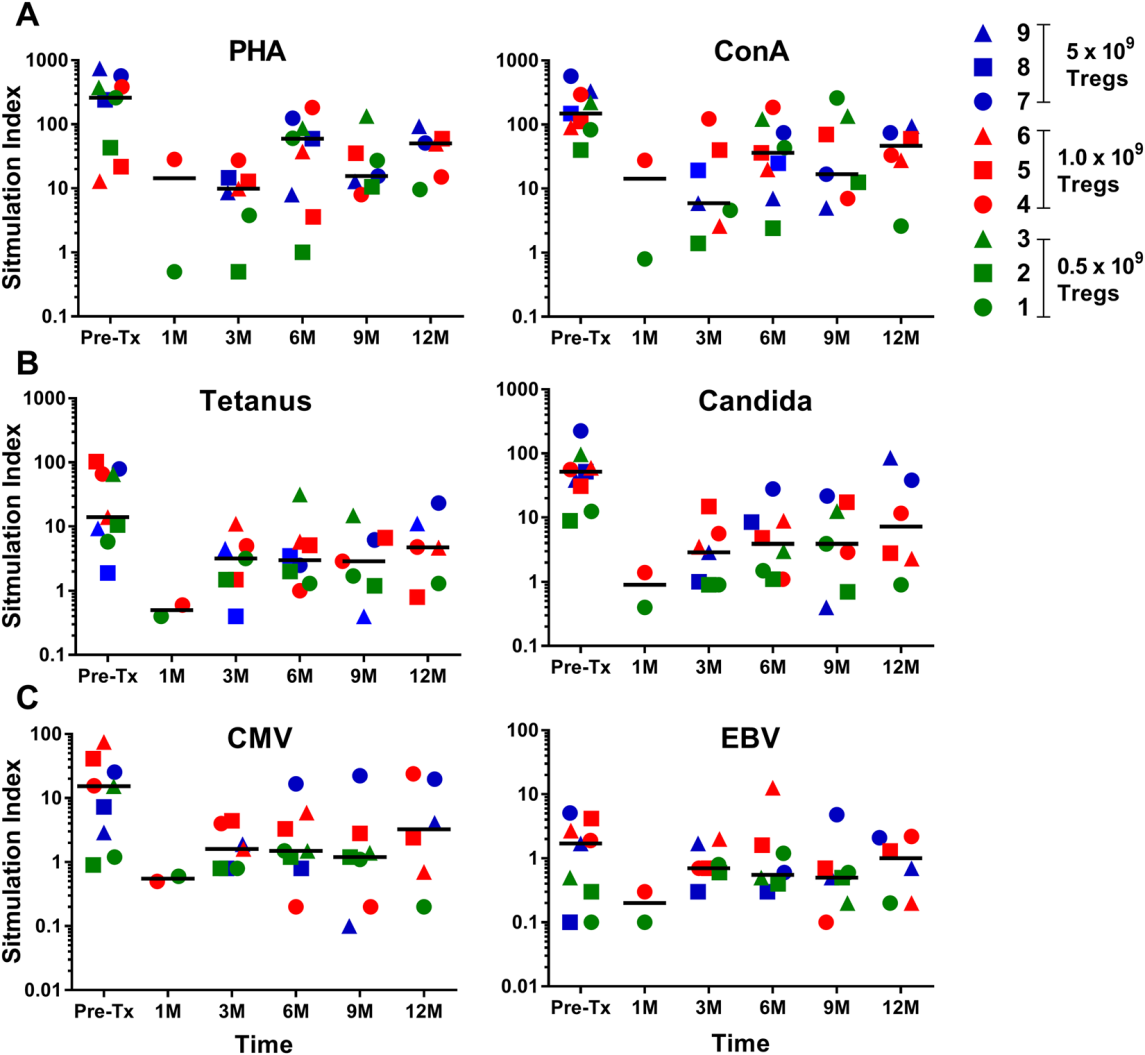
Recipient’s recall response to various mitogens and antigens: Stimulation indices calculated from the thymidine incorporation assays of recipient PBMCs plus antigen versus recipient PBMCs plus media only. Individual patient responses are displayed as symbols (n=9) and the grand median as –line at indicated time points post-transplant. Pre-Tx = pre-transplant.

We also examined the recipients’ recall responses to various antigens from pre-transplant to post-Treg infusion to 1 year post-transplant as a measure of their immunocompetence. We found the level of response to PHA, ConA, and CMV reached a nadir immediately post-transplant and gradually recovered even though they never reached the pre-transplant levels (Fig. 6) possibly due to the maintenance immunosuppression. However, it should be noted that no infections occurred in any patient within the year following transplant suggesting a maintained ability to respond to pathogenic antigens.

### Infusion of expanded Tregs into kidney transplant recipients is safe

A potential concern with the infusion of polyclonal expanded Tregs in lymphodepleted/immunosuppressed patients is the development of opportunistic infections and/or malignancies as a consequence of off-target Treg effects. In addition, the well-described plasticity of Tregs has raised concerns that infused cells might develop effector functions and cause rejection. There were no serious adverse events attributable to Treg infusion in any subject (Table 2); importantly, opportunistic infections associated with profound nonspecific immunosuppression, such as polyoma virus nephropathy and tissue invasive CMV disease did not occur in any of the phase 1 trial subjects. Protocol biopsies performed one month after Treg infusion (three months post-transplant) did not show rejection nor did recipients demonstrate donor specific antibody (DSA) development in peripheral blood. At 1 year post-transplant protocol biopsy, there was an episode of subclinical rejection with C4d deposition and de novo DSA development in one patient (subject 8); this was associated with IS non-compliance and was successfully treated with corticosteroids. Additionally subject #1, developed low titer DSA at 1 year post-transplant that was not associated with rejection on biopsy; this patient received the lowest cell tier and had experienced leukopenia requiring marked reduction in mycophenolate dosing making it difficult to determine if the low cell dose or the decrease in mycophenolate was the cause of DSA development. Additional follow up of subjects out to two years was notable for primary disease recurrence in one subject (#1). Patient and graft survival at two years is 100%. Overall, these results demonstrated that infusion up to 5 × 10^9^ polyclonally expanded recipient Tregs into renal transplant patients is safe.

**Table 2:** Clinical Outcomes of Treg cell infusion within renal transplant recipients.

Subject #	Date of		Cell # Administered (×10^9^)	Biopsy at		DSA at			Graft loss at	
	Transplant	Treg infusion		3 months	1 year	3 months	1 year	2 years	1 year	2 years
1	7/10/14	9/8/2014	0.5	NR	NR	−	+	+	NO	NO*
2	8/7/14	10/6/2014	0.5	NR	NR	−	−	−	NO	NO
3	8/29/14	10/28/2014	0.5	NR	NR	−	−	−	NO	NO
4	9/22/14	11/21/2014	1.0	NR	NR	−	−	−	NO	NO
5	12/4/14	2/2/2015	1.0	NR	NR	−	−	−	NO	NO
6	1/8/15	3/9/2015	1.0	NR	NR	−	−	−	NO	NO
7	2/2/2015	4/3/2015	5.0	NR	NR	−	−	−	NO	NO
8	3/27/2015	5/26/2015	5.0	NR	SCR C4d^+^	−	+	+	NO	NO
9	4/23/2015	6/22/2015	5.0	NR	NR	−	−	−	NO	NO

(NR) defined as no rejection;

(SCR) defined as subclinical rejection with C4d+ staining on biopsy.

(DSA) defined as donor specific antibodies.

*Developed recurrence of original disease in second year post transplant.

## Discussion

Patients that receive transplants are in desperate need of improved immune modulating options to control the alloresponse post-transplant. The long-term side effects of immunosuppressive medications has significantly tempered the short-term benefit of improved allograft survival. Therapeutic cell transfer in an effort to actively control the allogeneic immune response, allowing for minimization or complete elimination of drug based immunosuppression continues to be an unmet need. Initial studies in stem cell transplantation pioneered the way for adoptive Treg cell therapy as an alternative to classic immune suppression. We believe our study represents an important next step to the transition of Treg cell therapy into solid organ transplantation.

Treg cell therapy is still in its infancy with regards to commercialization, during this period of trial and error, there are considerable debates on a multitude of aspects. For example, how “pure” should the initial population be? Does that purity affect expansion? Potency? Efficacy? Purity can be defined differently across GMP cites and clinical centers but generally refers to a CD4^+^CD25^+^CD127^−^ phenotype. The only way conclusions and standards will begin to be made is for investigators and centers to share their experience and results. To that end, we utilized an immunomagnetic selection (without CD127 depletion) using the CliniMACS platform for the purification of the starting Tregs. This isolation procedure required only one CliniMACS Plus instrument and 6 hours of labor. Our initial product contained a minor population of CD127^+^ cells; however, with addition of sirolimus to the expansion media, the final Treg product contained negligible CD127^+^ cells (Fig. 2D). Other selection methods that include CD127 depletion use BD FACS Canto cell sorters (2 required) and over 20 hours of labor, resulting in a highly “pure” population. (Communications by Sandy Feng, ATC 2017 meeting, Chicago; and Qizhi Tang 3^rd^ Int. Workshop on Tolerance, Stanford, Sept. 8, 2017). We offer our selection and expansion protocol to the cell therapy community as a reproducible, cost-effective, streamlined approach toward commercialization whereas other methods may find trouble transferring their clinical protocols during the manufacturing scale up phase with industry partners. With purity still in mind and how it could affect product potency, we found our expanded Tregs demonstrated enhanced biologic activity, high FOXP3 expression and maintained epigenetic programming. These attributes were not part of the release criteria. However, their robustness is consistent with the notion that potential plasticity associated with Tregs might not be clinically relevant as FOXP3 expression is not inextricably linked with stability, consistent with the work of Sakaguchi *et al*^38,39^.

Previous studies have demonstrated that a high ratio of Tregs to conventional CD4^+^ T cells (Tconv), such as 1:1 or 1:2, may be needed to prevent transplant rejection^40,41^. A high prevalence of Tregs is, therefore, needed to thwart rejection by establishing a dominant tolerogenic milieu^35,42^. Considering the relatively small percentage of Tregs at normal steady state in peripheral blood (<5% of circulating CD4^+^ T cells), a drastic change of Tconv to Treg balance is achieved by, depletion of recipient alloreactive T cells and infusion of a sufficient number of expanded Tregs. As calculated by Tang *et al*. and ourselves, lymphodepletion using a T cell depleting monoclonal (Alemtuzumab) can reduce the CD4^+^ Tconv pool by 95–99% to 7.5 × 10^8^ − 4.6 × 10^9^ cells^43,44^. In our study, the infusion of the Tregs product was also deliberately delayed to allow for “wash out” of alemtuzumab to avoid the unintended destruction of the infused Tregs. In addition, conversion of patients from tacrolimus, which inhibits IL-2 production and theoretically would impair Treg survival, to sirolimus, a Treg-friendly IS agent, was completed before Treg infusion. Since this is a phase I trial we cannot conclude if Treg infusion was the reason why no adverse events or rejection events were observed, despite this deviation from our standard of care IS. However, we believe our approach allows for the best chance for Treg survival and enhanced potency post-infusion.

Only two other centers have successfully completed and published clinical trials utilizing expanded recipient Tregs in the context of solid organ transplantation^45^. Todo *et al*., have reported on the use of a “Treg-enriched” product generated in recipient anti-donor MLR in the presence of anti-CD80/86 monoclonal antibodies infused into liver recipients^46^. Lymphodepletion in subjects was achieved through the use of cyclophosphamide and splenectomy prior to Treg cell transfer. Immunosuppression was successfully weaned in 7 of 10 infused subjects; however, it should be noted that liver transplants are more tolerogenic than other transplants^47^. It is noteworthy that the “Treg enriched” cells infused by Todo *et al*. were comprised on average of 58.6% CD4^+^ and 16.9% CD8^+^ T cells with 24.8% of the CD4^+^ T cells expressing CD25^+^FOXP3^+^ Tregs. In contrast, our Treg product was composed on average of 98.8% CD4^+^CD25^+^, 0.7% CD8^+^, and ~80% FOXP3^+^ cells. The heterogeneous composition of the cell product utilized by Todo *et al*. raises questions as to what populations of cells infused are contributing to the ability to withdraw drug based immunosuppression. Furthermore, it remains unsettled as to what degree of purity of classical Tregs is minimally required for safe and efficacious clinical use. Ongoing work with therapeutic cell transfer using Tregs, such as work herein described, as well as that being conducted by the ONE Study (http://www.onestudy.org/), will hopefully provide additional insight in the near future. Recently, Chandran *et al.*^48^, reported on the UCSF experience with the infusion of 0.32 × 10^9^ autologous polyclonal Tregs in kidney recipients. Unlike our phase 1 trial which was the first to be reported and designed to evaluate Tregs as prophylaxis against allograft rejection, this UCSF study used Tregs, to treat subclinical inflammation observed on 6-month protocol biopsies. While only three patients were infused, no adverse infusion reactions or adverse events (treatment related) were observed. Importantly, their study demonstrated recipients that have already undergone transplantation and are on active immunosuppression can have Tregs collected, expanded, and re-infused, which may prove to be essential if additional doses of Tregs are needed for long term efficacy.

It is important to note that we did not observe any adverse clinical events (infection/rejection) following Treg administration at any of the doses used. This suggests that Treg cell therapy using polyclonal autologous Treg product is safe in the context of kidney transplantation. The robust state of lymphodepletion achieved with the conditioning regimen used in our clinical trial allowed to detect a profound and sustained increase in circulating numbers of Tregs in patients receiving Treg infusion. In contrast, contemporaneous historical control patients who received identical conditioning and maintenance IS showed no significant increase in circulating numbers of Tregs (Fig. 5D). Unfortunately the profound lymphodepletion due to the Alemtuzumab treatment also prevented us from a pre-Treg infusion immunophenotyping at 2 months, as the patients did not have sufficient cells in the allowable quantity of blood that could be drawn. We also did not employ methodologies to specifically label and track Tregs, such as deuterium labeling which has been described (47).

De novo DSA development was observed in two subjects in our clinical trial. This was associated with suboptimal immunosuppression due to drug intolerance in one subject and overt noncompliance in the other. DSA development has been associated with reduced long-term renal allograft survival^49,50^. Importantly, the de novo use of CNI free immunosuppressive regimens with mTOR inhibitors such as sirolimus or everolimus, or CNI conversion to mTOR inhibitor has been associated with an increased risk of developing DSA post-transplant^51,52^. A recent review concluded that risk of de novo DSA development described with mTOR inhibitor use can be eliminated through combination with reduced CNI exposure^53^. We have recently reported a prospective randomized study of a steroid free, low dose tacrolimus with everolimus regimen in kidney transplant recipients. Combined low dose tacrolimus and everolimus resulted in greater rejection-free graft survival than standard dose tacrolimus and mycophenolate mofetil. We did not see an increased risk of de novo DSA development in the mTOR arm. Importantly, the combined use of low dose tacrolimus and everolimus was associated with higher circulating numbers of Tregs, indicating this combination provides a favorable milieu for Treg survival (Traitanon O, *et al*. submitted). Such an mTOR – low dose CNI regimen may be well suited for future therapeutic cell transfer approaches utilizing Tregs.

In conclusion, we have verified our hypothesis that we can develop a protocol for the large-scale isolation and expansion of human Tregs and conduct a phase I clinical trial of Treg adoptive cell transfer therapy following kidney transplantation. This also sets the stage for a phase II clinical trial testing the efficacy of Treg infusion for tolerance induction or drug minimization. It remains to be determined whether Treg therapy, as a single immunomodulatory agent, is capable of inducing immunosuppression-free operational tolerance. Some adjunctive drug-based immunosuppression, or perhaps that combinatorial cell therapy with donor hematopoietic stem cell and Treg infusions maybe required.

## Methods

### Phase I clinical trial design and approval

The protocol was approved by the Northwestern Institutional Review Boards and the FDA (Clinical trial registration number 02145325 dated May 22, 2014; IND 15898). All procedures followed were in accordance with the ethical standards of the responsible committee on human experimentation (institutional and national) and with the Helsinki Declaration of 1975, as revised in 2008. Informed consent was obtained for all subjects. No organs/tissues were procured from prisoners and the organs were procured by Gift of Hope (https://www.giftofhope.org/) and the transplants were performed at the Comprehensive Transplant Center at Northwestern University. Subjects met institutional criteria as suitable living-donor kidney transplant recipients. The Phase 1 clinical trial protocol is provided in the Supplementary Addendum. Briefly, this was a nonrandomized study with 3 dosing tiers (0.5, 1, and 5 × 10^9^ cells infused/recipient,) with 3 recipients per tier. The clinical protocol is graphically shown in (Fig. 1A). Immunosuppression used included the use of induction antibody therapy in the form of alemtuzumab (Campath): 30 mg dose administered IV on day 0 (intraoperatively during kidney transplant) and on day +2 following kidney transplant. Corticosteroid induction (solumedrol 500 mg intravenously day 0, 250 mg day +1, and 125 mg day +2) was also employed; no maintenance oral corticosteroids were used. Maintenance immunosuppression consisted of 1) Mycophenolate (MPA; Myfortic): 720 mg–900 mg dosed twice daily orally starting Day −2 and continued post-transplant, 2) Tacrolimus (Prograf): dosed twice daily orally targeted to 12 hour trough concentrations of 8–12 ng/ml starting Day −2, continued until day +30 when conversion to sirolimus was initiated. Sirolimus (Rapamune) was dosed once daily, targeted to 24 hour trough concentrations of 8–12 ng/ml starting at Day +30 and then continued for the duration post-transplant. Conversion to sirolimus was completed before the infusion of expanded autologous Tregs which took place on Day +60 post-transplant. Protocol kidney biopsies were performed at 3 and 12 months post-transplant.

### Leukopheresis and Cryopreservation

Peripheral blood mononuclear cells (PBMCs) were collected from eligible patients by a single leukopheresis, collecting an average of 2.4 × 10^10^ mononuclear cells. The collected apheresis product was cryopreserved in 10% DMSO in normal saline using a rate controlled freezer. The cryopreserved product was stored in a monitored liquid nitrogen freezer.

### Thaw and Selection of CD25^+^ T-regulatory cells

Thawed apheresis product was washed and suspended in a de-clumping Buffer consisting of Miltenyi PBS/EDTA Buffer, 25% Human Serum Albumin (HSA), MgCl_2,_ and Pulmozyme (Genentech). CD8 and CD19 depletion of the apheresis product, and subsequent CD25^+^ enrichment was carried out on the CliniMACS Plus using GMP reagents and protocols (Miltenyi). The resulting product was used to expand as the Tregs.

### Expansion of Regulatory T cells

Growth media (GM) consisted of TexMACS medium supplemented with 5% heat-inactivated AB serum, 1,000 IU/ml IL-2, 100 ng/ml Sirolimus (SRL: Rapamycin; Sigma Aldrich) and 1 µg/ml TGF-β (Miltenyi). G-Rex bioreactor (Wilson Wolf,) cultures were initiated with 2 × 10^7^ to 3 × 10^7^ cells and Exp-Act® beads at a 4:1 bead: cell ratio. GM was added every two to three days to maintain a cell density of 1 × 10^6^/mL. On Day 7, the cells were restimulated with Exp-Act® beads, at a ratio of 1:1 beads. On Day 14, in-process testing was preformed: 14-day aerobic and anaerobic sterility cultures, 30-day fungal culture, mycoplasma, endotoxin detection, cell counts, viability, phenotyping, and the Treg suppression assay. SRL was not added after Day 9 and cells were harvested on Day 21.

### Exp-Act® beads removal and product safety testing

The harvested Treg product was processed on the CliniMACS Plus instrument to remove Exp-Act® beads. The eluted product was evaluated for residual Exp-Act® beads using flow cytometry. Final product safety testing included all tests performed on Day 14 with the addition of residual bead count. (Table 1).

### Flow Cytometry

For research expansion samples on days 0, 14 and 21 as well as post-transplant immune monitoring, antibodies against CD4-FITC, CD127-PE, CD3-ECD, CD25-PC7 (all Beckman-Coulter) and FOXP3-PC5 (eBioscience, San Diego, CA) were used. Chemokine and receptor characterizations were performed using CXCR3/GARP-Pacific Blue, CXCR4/CD62L-PerCP-Cy5.5, CCR7/CD45RO-APC-Cy7, CD45RA-Alexa700, and CTLA-4-APC (all from eBioscience, San Diego, CA).

For the GMP expanded Treg products 1–9, three separate panels were used consistently throughout Treg expansion and all antibodies were purchased from Miltenyi. Panel one: CD8-APC, CD20-FITC, CD14/15/56-PE, CD45-Pacific Blue and 7AAD-PerCP-Cy5.5. Panel two: CD25-APC, CD4-FITC, CD127-PE, CD45-Pacific Blue and 7AAD-PerCP-Cy5.5. Panel three: CD4-FITC, CD25-PE, Foxp3-APC and CD45-Pacific Blue. Research grade flow cytometry was performed on a Beckman-Coulter FC500 whereas clinical flow cytometry was done on a BD-LSR, as previously described^37,54,55^.

Patient post-transplant flow cytometric analyses were done on a Beckman-Coulter FC500 using antibodies (all Beckman-Coulter) against CD4-FITC, CD127-PE, CD3-ECD, CD25-PC7 and FOXP3-PC5 (eBioscience); IL10-FITC, IgD/M-PE, CD19-ECD, CD27-PC5, CD24-PC7; Helios-FITC, CD3-ECD, CD28-PC5, CD8-PC7 and FOXP3-PE (eBioscience), among others.

### Treg suppression assay

The responder cells consisted of cryopreserved CD8^+^CD19^+^CD4^+^CD25^-^ cells obtained from pooled non-selected leukopheresis product fractions of Treg isolation, autologous to the Tregs (R-PBMC). They were stimulated with fresh allogeneic third party donor (I-PBMC) at a ratio of 1:1 in U-bottom 96-well plates in triplicate. Tregs or irradiated R-PBMC (control) were added at indicated Treg: Responder ratios at the initiation of the mixed lymphocyte reaction (MLR) suppression assays. After 5 days, ^3^H-thymidine (1uCi/well) was added to the cultures during the final 16–20 hours and incorporation of ^3^H-thymidine was used to measure proliferation. The MLR inhibition was quantified as previously described^56,57^:

$$ \% \,{\rm{inhibition}}=1-(\frac{\mathrm{Proliferation}\,\,{\rm{in}}\,{\rm{presence}}\,{\rm{of}}\,{\rm{Tregs}}-{\rm{back}}\,{\rm{response}}\,{\rm{by}}\,{\rm{Tregs}}}{{\rm{Proliferation}}\,{\rm{in}}\,{\rm{presence}}\,{\rm{of}}\,{\rm{Rx}}\,{\rm{controls}}-{\rm{back}}\,{\rm{response}}\,{\rm{by}}\,{\rm{Rx}}\,{\rm{controls}}})\times 100$$

### Treg Infectious tolerance assay

The ability of expanded Tregs to generate new Tregs infectiously from naïve responder cells was measured using the “Treg-MLR” as described previously^37^. Recipient CFSE-labeled responder cells (R-PBMC) were stimulated with irradiated and PKH26-labelled I-PBMC in presence of PKH-labelled Tregs. On day 7, flow cytometry was performed on the cultured cells after labeling with CD127-PE, CD4-ECD, CD25-PC7, and FOXP3-PC5. PKH26-labelled modulators were gated out and CD4^+^ cells that proliferated were analyzed for CD25 and FOXP3 expressions.

### Methylation Analysis

Cryopreserved Treg cell pellet were evaluated by EpigenDx for DNA extraction, bisulfite conversion, and pyrosequencing. Percent methylation was calculated as % methylated cytosine/ (% methylated cytosine + unmethylated cytosine).

### TCRβ repertoire analysis

Expanded Tregs and apheresis cell pellets of 0.5 × 10^6^ were sent to Adaptive Biotechnologies (Seattle, WA) for survey level TCRβ sequencing. Analyses of the sequencing data including Clonality index and number of productive and unique sequencing reads were done using algorithms developed by Adaptive Biotechnologies.

### Mitogen and recall response assay

Lymphoproliferative responses of 1 × 10^5^ recipient PBMCs were determined using optimized concentrations of mitogens and recall antigens using standard radioactive ^3^H-TdR incorporation assays as previously described^55^. Stimulation indices (SI) were calculated using the formula: CPM in Experimental Combinations / CPM in Unstimulated Controls.

### Statistical Analysis

Paired Student T-tests and Wilcoxon signed rank tests for parametric and nonparametric calculations respectively were used. P values of ≤0.05 were considered statistically significant and are plotted as dotted lines in the Figures.

## Electronic supplementary material

Supplementary Files

## References

[1] Eggers PW (1988). Effect of transplantation on the Medicare end-stage renal disease program. N Engl J Med.

[2] Hariharan S (2000). Improved graft survival after renal transplantation in the United States, 1988 to 1996. N Engl J Med.

[3] Barry JM (1992). Immunosuppressive drugs in renal transplantation. A review of the regimens. Drugs.

[4] Suthanthiran M, Strom TB (1994). Renal transplantation. N Engl J Med.

[5] Helderman JH, Van Buren DH, Amend WJ, Pirsch JD (1994). Chronic immunosuppression of the renal transplant patient. J Am Soc Nephrol.

[6] Gaston RS (2001). Maintenance immunosuppression in the renal transplant recipient: an overview. Am J Kidney Dis.

[7] Pirsch JD (1992). Hyperlipidemia and transplantation: etiologic factors and therapy. J Am Soc Nephrol.

[8] Shaw LM, Kaplan B, Kaufman D (1996). Toxic effects of immunosuppressive drugs: mechanisms and strategies for controlling them. Clin Chem.

[9] Boubenider S (1997). Incidence and consequences of post-transplantation lymphoproliferative disorders. J Nephrol.

[10] Fishman JA, Rubin RH (1998). Infection in organ-transplant recipients. N Engl J Med.

[11] DeMario MD, Liebowitz DN (1998). Lymphomas in the immunocompromised patient. Semin Oncol.

[12] Sia IG, Paya CV (1998). Infectious complications following renal transplantation. Surg Clin North Am.

[13] Pirsch JD, Miller J, Deierhoi MH, Vincenti F, Filo RS (1997). A comparison of tacrolimus (FK506) and cyclosporine for immunosuppression after cadaveric renal transplantation. FK506 Kidney Transplant Study Group. Transplantation.

[14] Wood, K. J. *et al*. Regulatory cells in transplantation. *Novartis Found Symp* **252**, 177–188, discussion 188–193, 203–110 (2003). 14609219

[15] Xia G, He J, Leventhal JR (2008). *Ex vivo*-expanded natural CD4+ CD25+ regulatory T cells synergize with host T-cell depletion to promote long-term survival of allografts. Am J Transplant.

[16] Xia G, He J, Zhang Z, Leventhal JR (2006). Targeting acute allograft rejection by immunotherapy with *ex vivo*-expanded natural CD4+ CD25+ regulatory T cells. Transplantation.

[17] Heinrichs J (2016). Regulatory T-Cell Therapy for Graft-versus-host Disease. J Immunol Res Ther.

[18] Heinrichs J (2016). CD8(+) Tregs promote GVHD prevention and overcome the impaired GVL effect mediated by CD4(+) Tregs in mice. Oncoimmunology.

[19] Li J (2015). HY-Specific Induced Regulatory T Cells Display High Specificity and Efficacy in the Prevention of Acute Graft-versus-Host Disease. J Immunol.

[20] Semple K, Yu Y, Wang D, Anasetti C, Yu XZ (2011). Efficient and selective prevention of GVHD by antigen-specific induced Tregs via linked-suppression in mice. Biol Blood Marrow Transplant.

[21] Trzonkowski P (2009). First-in-man clinical results of the treatment of patients with graft versus host disease with human *ex vivo* expanded CD4+ CD25+ CD127− T regulatory cells. Clin Immunol.

[22] Brunstein CG (2011). Infusion of *ex vivo* expanded T regulatory cells in adults transplanted with umbilical cord blood: safety profile and detection kinetics. Blood.

[23] Di Ianni M (2011). Tregs prevent GVHD and promote immune reconstitution in HLA-haploidentical transplantation. Blood.

[24] Bluestone JA (2015). Type 1 diabetes immunotherapy using polyclonal regulatory T cells. Sci Transl Med.

[25] Leventhal J (2012). Chimerism and tolerance without GVHD or engraftment syndrome in HLA-mismatched combined kidney and hematopoietic stem cell transplantation. Sci Transl Med.

[26] Levitsky J (2011). Allospecific Regulatory Effects of Sirolimus and Tacrolimus in the Human Mixed Lymphocyte Reaction. Transplantation.

[27] Gallon L (2015). Differential Effects of Calcineurin and Mammalian Target of Rapamycin Inhibitors on Alloreactive Th1, Th17, and Regulatory T Cells. Transplantation.

[28] Kim KW, Chung BH, Kim BM, Cho ML, Yang CW (2015). The effect of mammalian target of rapamycin inhibition on T helper type 17 and regulatory T cell differentiation *in vitro* and *in vivo* in kidney transplant recipients. Immunology.

[29] Peccatori J (2015). Sirolimus-based graft-versus-host disease prophylaxis promotes the *in vivo* expansion of regulatory T cells and permits peripheral blood stem cell transplantation from haploidentical donors. Leukemia.

[30] Schmidt A, Eriksson M, Shang MM, Weyd H, Tegner J (2016). Comparative Analysis of Protocols to Induce Human CD4+ Foxp3+ Regulatory T Cells by Combinations of IL-2, TGF-beta, Retinoic Acid, Rapamycin and Butyrate. PLoS ONE [Electronic Resource].

[31] Sawitzki B (2001). Regulatory tolerance-mediating T cells in transplantation tolerance. Transplant Proc.

[32] Lee I (2005). Recruitment of Foxp3+ T regulatory cells mediating allograft tolerance depends on the CCR4 chemokine receptor. J Exp Med.

[33] Zhang N (2009). Regulatory T cells sequentially migrate from inflamed tissues to draining lymph nodes to suppress the alloimmune response. Immunity.

[34] Graca L, Cobbold SP, Waldmann H (2002). Identification of regulatory T cells in tolerated allografts. J Exp Med.

[35] Francis RS (2011). Induction of transplantation tolerance converts potential effector T cells into graft-protective regulatory T cells. Eur J Immunol.

[36] Gershon RK, Kondo K (1971). Infectious immunological tolerance. Immunology.

[37] Levitsky J (2009). The human “Treg MLR”: immune monitoring for FOXP3+ T regulatory cell generation. Transplantation.

[38] Ohkura N (2012). T cell receptor stimulation-induced epigenetic changes and Foxp3 expression are independent and complementary events required for Treg cell development. Immunity.

[39] Sakaguchi S, Miyara M, Costantino CM, Hafler DA (2010). FOXP3+ regulatory T cells in the human immune system. Nat Rev Immunol.

[40] Hara M (2001). IL-10 is required for regulatory T cells to mediate tolerance to alloantigens *in vivo*. J Immunol.

[41] Graca L (2002). Both CD4(+)CD25(+) and CD4(+)CD25(−) regulatory cells mediate dominant transplantation tolerance. J Immunol.

[42] Kendal AR (2011). Sustained suppression by Foxp3+ regulatory T cells is vital for infectious transplantation tolerance. J Exp Med.

[43] Tang Q, Lee K (2012). Regulatory T-cell therapy for transplantation: how many cells do we need?. Curr Opin Organ Transplant.

[44] Mathew JM (2018). Generation and Characterization of Alloantigen-Specific Regulatory T Cells For Clinical Transplant Tolerance. Scientific Reports.

[45] Todo S (2016). A pilot study of operational tolerance with a regulatory T-cell-based cell therapy in living donor liver transplantation. Hepatology.

[46] Todo, S. & Yamashita, K. Anti-donor regulatory T cell therapy in liver transplantation. *Hum Immunol* **79**, 10.1016/j.humimm.2017.12.010 (2018). 10.1016/j.humimm.2017.12.010 29292027

[47] Knechtle SJ, Kwun J (2009). Unique aspects of rejection and tolerance in liver transplantation. Semin Liver Dis.

[48] Chandran S (2017). Polyclonal Regulatory T Cell Therapy for Control of Inflammation in Kidney Transplants. Am J Transplant.

[49] Wiebe C (2012). Evolution and clinical pathologic correlations of de novo donor-specific HLA antibody post kidney transplant. Am J Transplant.

[50] Everly MJ (2013). Incidence and impact of de novo donor-specific alloantibody in primary renal allografts. Transplantation.

[51] Rostaing L (2015). Fibrosis progression according to epithelial-mesenchymal transition profile: a randomized trial of everolimus versus CsA. Am J Transplant.

[52] Liefeldt L (2012). Donor-specific HLA antibodies in a cohort comparing everolimus with cyclosporine after kidney transplantation. Am J Transplant.

[53] O’Leary JG (2016). The Influence of Immunosuppressive Agents on the Risk of De Novo Donor-Specific HLA Antibody Production in Solid Organ Transplant Recipients. Transplantation.

[54] Leventhal JR (2015). Interim Results of a Phase 1 Trial of Treg Adoptive Cell Transfer (TRACT) in Living Donor Kidney Transplant Recipients. Am J Transplant.

[55] Leventhal JR (2016). Nonchimeric HLA-Identical Renal Transplant Tolerance: Regulatory Immunophenotypic/Genomic Biomarkers. Am J Transplant.

[56] Mathew JM (2000). Donor bone marrow-derived chimeric cells present in renal transplant recipients infused with donor marrow. I. Potent regulators of recipient antidonor immune responses. Transplantation.

[57] Levitsky, J., *et al* Inhibitory Effects of Belatacept on Allospecific Regulatory T-Cell Generation in Humans. *Transplantation* **96**, 689–696, 10.1097/TP.1090b1013e31829f31607 (2013). 10.1097/TP.0b013e31829f1607 PMC3800494 23883971

