# Supplementary material for: A Phase I Clinical Trial with Ex Vivo Expanded Recipient Regulatory T cells in Living Donor Kidney Transplants
Source: Sci Rep. 2018 May 9;8:7428. doi: 10.1038/s41598-018-25574-7 (PMC5943280; doi:10.1038/s41598-018-25574-7)
Supplement: Supplementary file 1 — Supplementary Files [file 41598_2018_25574_MOESM1_ESM.pdf]

A Phase I Clinical Trial with *Ex Vivo* Expanded Recipient Regulatory T cells in Living Donor Kidney Transplants

James. M. Mathew, Jessica. H.-Voss, Ann LeFever, Iwona Konieczna, Cheryl Stratton, Jie He, Xuemei Huang, Lorenzo Gallon, Anton Skaro, Mohammed Javeed Ansari, Joseph R. Leventhal.

SUPPLEMENTAL FIGURES:

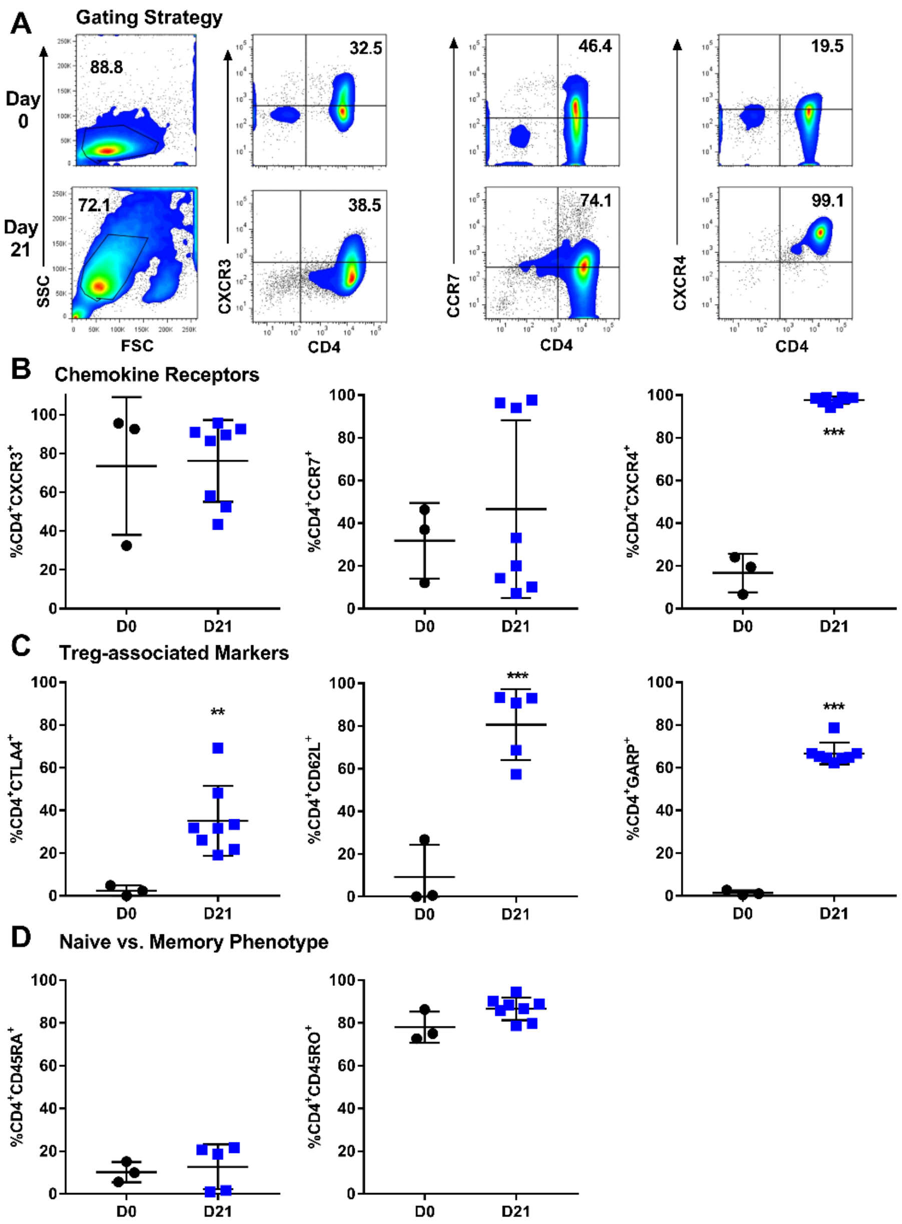

Fig. S1: Characterization of cell surface markers on Tregs post expansion.

(A) Representative gating strategy for phenotyping Tregs: analysis was performed on all live cells for all markers, FSC^{+}SSC^{+}. These analysis were performed on research-grade expanded Tregs cultured under comparable conditions as those infused into patients.

(B-D) Average (± SD) of percentage of CD4^{+} cells expressing indicated key chemokine receptors, Treg-associated markers, and naïve vs. memory phenotype from day 0 (n=3) to day 21 (n=5) of expansion culture. **p<0.01; *** p<0.005.

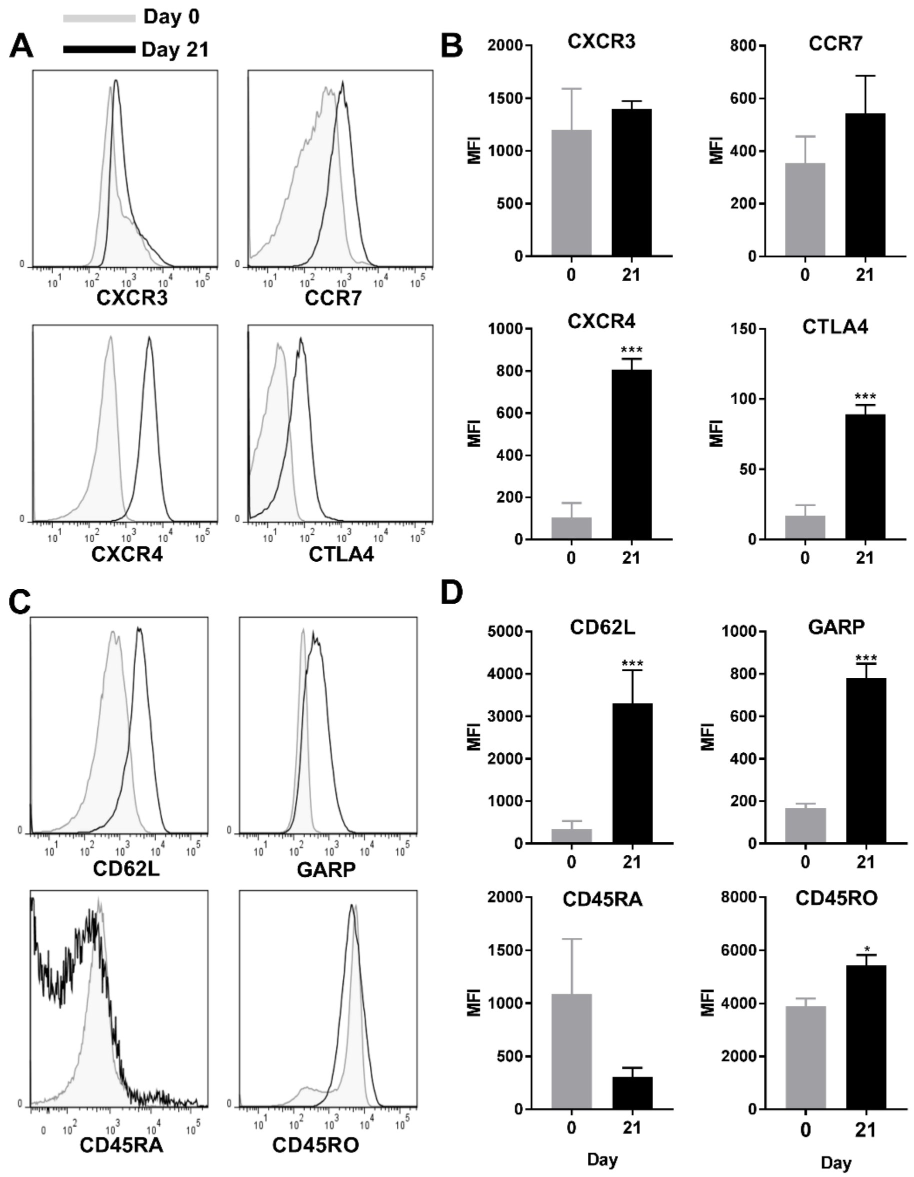

Fig. S2: Changes in the intensity of cell surface receptor expression post expansion:

(A and C) Representative histogram shift in receptor expression on Treg cell surface from Day 0 to Day 21 of expansion.

(B and D) Quantitative mean fluorescent intensity changes (average +SD) of surface receptor expression throughout expansion protocol. n= (5). *p<0.05; ***p<0.005.

A

	Average Total Rearrangements	Average Productive Rearrangements	Average % Productive
**Day 0 Apheresis**	60336	48491	80
**Day 21 Tregs**	76115	62363	82

B

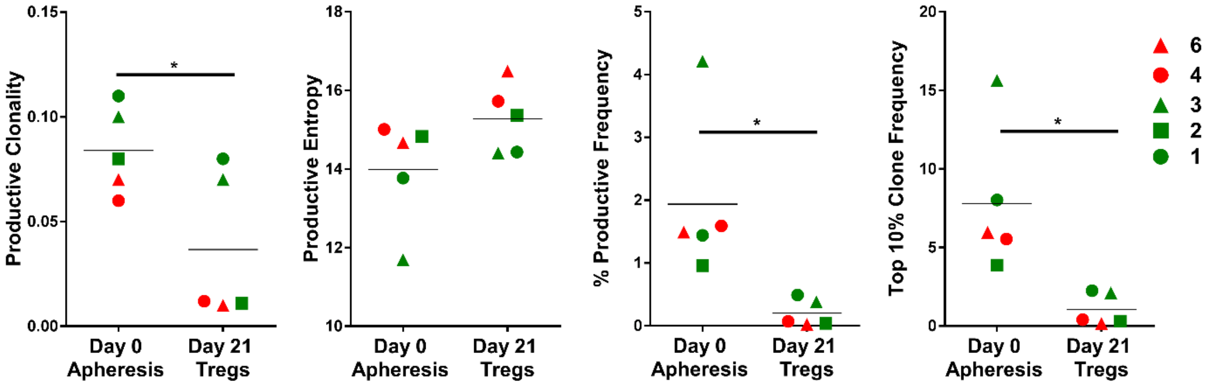

Fig. S3: T cell receptor diversity is maintained post Treg expansion:

(A) Table depicting the average (n=5) "rearrangements" defined as a unique nucleotide sequence resulting from V, D, J gene rearrangements; "productive" defined as the count of those unique rearrangements that are in-frame and can produce a functional protein receptor.

(B) Only Productive values are considered due to definition defined in (A). Clonality; defined as the inverse of Shannon's entropy where values near 1 represent a sample with monoclonal T cell repertoire and values near 0 represent a highly diverse T cell repertoire. Entropy; defined as Shannon's entropy where higher value samples represent greater diversity and lower value samples will share more nucleotide sequences. Top 10% clone frequency; defined as the frequency of the top 10 productive rearrangements among all rearrangements. All analysis was performed using ImmunoSEQ 3.0 analyzer and all definitions provided by Adaptive Biotechnologies. *$p<0.05$.

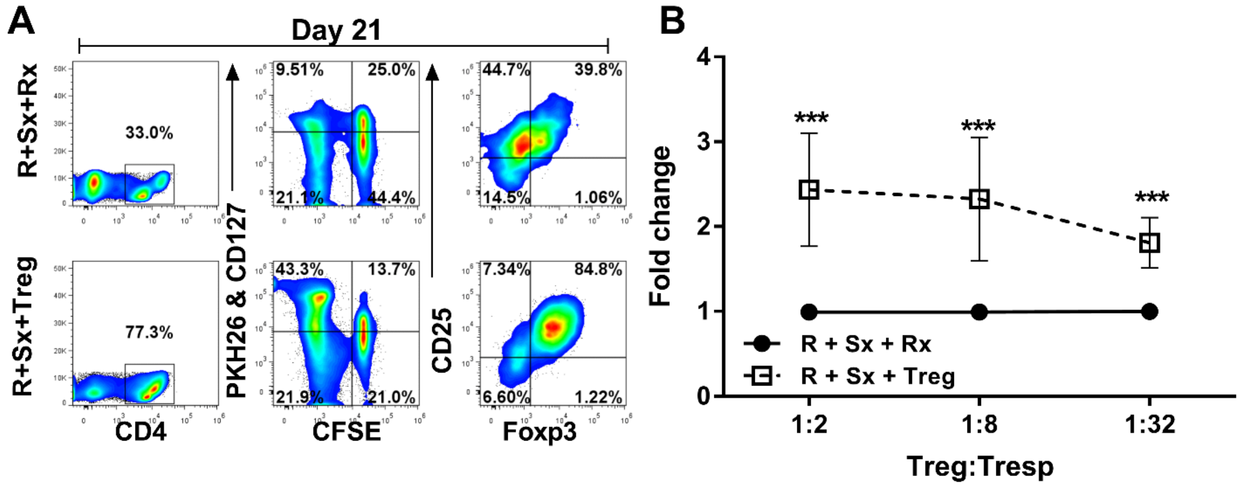

Fig. 4: In process infectious tolerance testing of Tregs:

(A) Representative flow gating strategy for the Treg-MLR to identify responder cells (R) proliferating (CFSE-diluted) and becoming Tregs ($CD25^+FOXP3^+$) in the presence (bottom) or absence (top) of expanded Tregs. Sx denotes irradiated healthy allogeneic PBMC's (stimulators); (Rx) denotes irradiated responder cells used as a control to keep cell number consistent across groups.

(B) Average (±SD) of fold change in percent of newly generated Tregs ($CD4^+CD127^-PKH26^-$ $CD25^+FOXP3^+$ cells in cultures when Tregs were present (R+ Sx + Treg) divided by when Tregs were absent (R+ Sx+ Rx) in CFSE-diluted fraction of the responding R cells in the Treg-MLR (n=8), ***p<0.005.

Auxiliary Supplementary Materials S1: Approved IRB Protocol and Trial Design

Northwestern University
Feinberg School of Medicine
Division of Organ Transplantation

"A PHASE I, SINGLE CENTER TRIAL OF ADOPTIVE THERAPY WITH TREG ADOPTIVE CELL TRANFSER (TRACT) TO PREVENT REJECTION IN LIVING DONOR KIDNEY TRANSPLANT RECIPIENTS"

**Principal Investigator:**	Anton Skaro, MD, PhD<br>Department of Surgery/Division of Organ Transplantation 676 N. St. Clair Street, Suite 1900 Arkes Pavilion Chicago. IL 60611
**Co-Investigators:**	Joseph Leventhal, MD, PhD<br>Lorenzo Gallon, MD<br>Ann LeFever, PhD<br>James Mathew, PhD
**Funding Sponsor:**	Northwestern University
**Study Products:**	Autologous, ex-vivo expanded regulatory T cells (Tregs)
**IND Sponsor: IND Number:**	Joseph Leventhal, MD, PhD<br>15898
**IND Title:**	Natural, Regulatory T Cells (T-Regs), Expanded with Interleukin-2, Exp-Act Beads® (CD3/CD28; Miltneyi), Rapamycin and Transforming Growth Factor β

Version 3.00

Date: V1_1-8-14
Amended: V2_3-27-14
Amended: V3_2-2-2015
Administrative Change:

Table of Contents

STUDY SUMMARY 1
1 INTRODUCTION 3
1.1 BACKGROUND 4
1.2 INVESTIGATIONAL AGENT 4
1.3 PRECLINICAL DATA IN SUPPORT OF ADOPTIVE THERAPY WITH REGULATORY T CELLS (TRACT) 6
1.4 CLINICAL DATA TO DATE 7
1.5 CLINICALLY APPLICABLE STRATEGY FOR EX VIVO EXPANSION OF NTREGS 8
1.6 THE IMPORTANCE OF CONVENTIONAL T CELL DEPLETION TO LEVERAGE THE THERAPEUTIC EFFICACY OF TREGS 9
1.7 EXPERIENCE WITH EXPANSION OF HUMAN TREGS 9
1.8 MATHEWS CENTER FOR CELLULAR THERAPY (MCCT) AT NORTHWESTERN MEMORIAL HOSPITAL 10
1.9 APPROACH 10
2 STUDY DRUGS 11
2.1 STANDARD OF CARE PHARMACOLOGIC AGENTS USED IN KIDNEY TRANSPLANTATION 11
2.2 STUDY DRUG 14
2.3 DOSE RATIONALE 16
3 STUDY OBJECTIVES 17
3.1 PRIMARY OBJECTIVE 17
3.2 PRIMARY OUTCOME MEASURES 17
3.3 SECONDARY OBJECTIVES 17
4 STUDY DESIGN 18
4.1 GENERAL DESIGN 18
4.2 PRIMARY SAFETY ENDPOINT 20
5 SUBJECT SELECTION 20
5.1 INCLUSION CRITERIA 20
5.2 EXCLUSION CRITERIA 20
5.3 SUBJECT RECRUITMENT AND SCREENING 21
5.3.1 Subject Information and Consent 21
5.3.2 Avoidance of Coercion 21
5.4 EARLY WITHDRAWAL OF SUBJECTS 22
5.4.1 When and How to Withdraw Subjects 22
5.4.2 Data Collection and Follow-up for Withdrawn Subjects 22
6 STUDY RELATED THERAPIES 23
6.1 STANDARD OF CARE IMMUNOSUPPRESSION 23
6.2 TRACT CELLULAR PRODUCT (INVESTIGATIONAL DRUG) 23
6.2.1 Leukopheresis 23
6.2.2 cGMP Manufacture 24
6.2.3 Expanded autologous TRACT Infusion 255
6.3 TREATMENT OF ACUTE REJECTION 25
6.4 CRITERIA FOR CONVERSION BACK TO TACROLIMUS 25
6.5 CRITERIA FOR ADDITION OF ORAL CORTICOSTEROIDS 26
6.6 PREPARATION AND ADMINISTRATION OF STUDY DRUGS 26
6.6.1 TRACT (Investigational Product) 26
6.6.2 Subject Compliance Monitoring 26
6.6.3 Prior and Concomitant Therapy 26
6.6.4 Packaging 27
7 STUDY PROCEDURES 27
7.1 SAFETY 27

7.2 INFECTIOUS EPISODES 27
7.3 CLINICAL AND LABORATORY ASSESSMENTS 27
7.3.1 Pre-Transplant *27*
7.3.2 Post-Transplant *28*
7.3.3 Core Renal Biopsies *28*
7.4 DOSE LIMITING TOXICITY (DLT) DETERMINATION IN THE STUDY 28
7.5 STOPPING RULES 30
7.6 DATA ANALYSIS 30
7.6.1 Analysis of the Primary Endpoints *30*
7.6.2 Analysis of Secondary Endpoints *31*

8 DATA SAFETY MONITORING PLAN 31

8.1 MONITORING AND QUALITY ASSURANCE 31

9 SAFETY AND ADVERSE EVENTS 32

9.1 DEFINITIONS 32
9.2 RECORDING OF ADVERSE EVENTS 35
9.3 REPORTING OF SERIOUS ADVERSE EVENTS 35
9.3.1 Notification by Investigator-Sponsor *35*
9.4 MEDICAL MONITORING 36

10 DATA HANDLING AND RECORD KEEPING 36

10.1 CONFIDENTIALITY 36
10.2 SOURCE DOCUMENTS 37
10.3 CASE REPORT FORMS 37
10.4 RECORDS RETENTION 37
10.5 AUDITING AND INSPECTING 38

11 ETHICAL CONSIDERATIONS 38
12 STUDY FINANCES 38

12.1 FUNDING SOURCE 38
12.2 CONFLICT OF INTEREST 38
12.3 SUBJECT STIPENDS OR PAYMENTS 39

13 PUBLICATION PLAN 39

13.1 SUBJECT DATA PROTECTION 39

14 REFERENCES 39
15 ATTACHMENTS 42

List of Abbreviations

ABO	Blood groups A, B and O
AE	Adverse Event
ALT	Alanine Aminotransferase
AST	Aspartamine Amino transferase
Bicarb	Bicarbonate
BID	Twice Daily
BUN	Blood Urea Nitrogen
Cl	Chloride
CMV	Cytomegalovirus
CNI	Calcineurin Inhibitor
CRF	Case Report Form
CS	Corticosteroids
CsA	Cyclosporine
D	Donor
EBV	Epstein Barr Virus
ESRD	End Stage Renal Disease
FDA	Food and Drug Administration
GCP	Good Clinical Practice
HCG	Human chorionic gonadotropin
HLA	Human Leukocyte Antigen
HPLC	High pressure liquid chromatography
ICH	International Conference on Harmonization
IL-2	Interleukin-2
IRB	Institutional Review Board
K	Potassium
LDH	Lactate Dehydrogenase
LURD	Living Unrelated Donor
LRD	Living Related Donor
MLR	Mixed lymphocyte reaction
MyF	Myfortic
Na	Sodium
-	Negative
POD	Post Operative Day
PRA	Panel Reactive Antibody
PT	Prothrombin Time
PTT	Partial Thromboplastin Time
+	Positive
R	Recipient
Rapa	Rapamune (Sirolimus)
SAE	Serious Adverse Event
S.D.	Standard Deviation
TAC	Tacrolimus
T/B	T-cells and B-cells
Treg	Regulatory T cells
nTreg	Naturally occurring regulatory T cells

Study Summary

Title	***"A PHASE I, SINGLE CENTER TRIAL OF ADOPTIVE THERAPY WITH TREG ADOPTIVE CELL TRANSFER (TRACT) TO PREVENT REJECTION IN LIVING DONOR KIDNEY TRANSPLANT RECIPIENTS***
Short Title	Regulatory T cell infusion in Kidney Transplant Recipients
Phase	Clinical study phase I
Methodology	A single-center, open-label, dose-finding safety trial.
Study Duration	60 weeks
Study Center(s)	Single-center – Northwestern University
Objectives	Primary: Determine the safety of autologous, expanded Treg adoptive cell transfer (TRACT) administered to living donor renal transplant recipients.
Number of Subjects	12
Diagnosis and Main Inclusion Criteria	Subjects with end-stage renal disease (ESRD) who undergo a living donor kidney transplant
Study Products	Autologous, ex vivo expanded natural regulatory T cells (Tregs)
Duration of administration	Single infusion of expanded Tregs day +60 following living donor kidney transplantation
Statistical Methodology	Mann-Whitney, Fisher test, Student's t test, Anova and Chi-Squared analyses will be used to analyze the safety data as needed.

ESTIMATED ENROLLMENT: 12
STUDY START DATE: MARCH 2014
ESTIMATED STUDY COMPLETION: MARCH 2016
ESTIMATED PRIMARY COMPLETION DATE: APRIL 2015

TREATMENT SCHEMA — ASSIGNED INTERVENTIONS

EXPERIMENTAL: TRACT (N=12) — BIOLOGIC: EX VIVO, EXPANDED AUTOLOGOUS TREG INFUSION

Kidney transplantation using standard of care immunosuppressive agents will be performed, followed by intravenous infusion of autologous, expanded Tregs (TRACT) at day 60 after transplantation. Subjects will be administered only one dose of Treg at the specified dose level. Cell infusions will be escalated in the absence of adverse events within 30 days of infusion using the following schema:

Tier 1: 0.5 x 10^9 total expanded nTregs (n=3)
Tier 2: 1 x 10^9 total expanded nTregs (n=3)
Tier 3: 5 x 10^9 total expanded nTregs (n=3)
Tier 4: 8 x 10^9 total expanded nTregs (n=3)

Patients will undergo a non-mobilized leukopheresis for isolation of Tregs at least one week but no more than one year before scheduled date of renal transplant. The leukopheresis product will be cryopreserved for later expansion and administration. The Treg final product will not be cryopreserved and will be administered after 21 days of ex vivo expansion upon meeting required release criteria.

Immunosuppression:

Alemtuzumab (Campath): 30mg dose administered IV day 0 and day +2 following kidney transplant

Mycophenolate (MPA) (Myfortic): 720mg-900mg dosed twice daily orally starting Day -2 until withdrawal or end of study

Tacrolimus (Prograf): Oral twice daily dose targeted to trough concentration of 8-12ng/ml starting Day -2, continued until day +30 when conversion to Sirolimus will be conducted

Sirolimus (Rapamune): Oral daily dose targeted to trough concentration of 8-12ng/ml starting at Day +30 until withdrawal or end of study

Procedures: Leukopheresis, Living Donor Kidney Transplantation, Treg infusion

Investigator Signature Page

This document is a protocol for a human research study. This study is to be conducted according to US and international standards of Good Clinical Practice (FDA 21 CFR312 and International Conference on Harmonization guidelines), applicable government regulations and Institutional research policies and procedures.

By signing below, I agree to conduct the research by US and International Standards of GCP FDA 21 CFR312 and ICH guidelines. Being the principal investigator, I also agree to oversee that the subinvestigators and all research staff abide by the same standards and principles.

____

Anton Skaro, MD, PhD Date

1 Introduction

Transplantation is the treatment of choice for most causes of end stage renal disease. (1, 2) However, without some modification of the recipient's immune system all allografts succumb to rejection. To prevent this, patients must take immunosuppressive drugs for life, generally a combination of steroids, a calcineurin inhibitor (CNI), such as cyclosporine or tacrolimus, and an antiproliferative agent (azathioprine, mycophenolate mofetil or Sirolimus) (3-6). Induction with a brief course of an anti-T lymphocyte antibody preparation (daclizumab, basiliximab, muromonab, alemtuzumab, polyclonal anti-thymocyte globulin) is also used in approximately 70% of U.S. transplant centers.

Dependence on immunosuppression tempers the substantial benefit obtained from transplantation (1-13). The typical regimens are relatively complex and expensive. More importantly, they increase the risk of opportunistic infection and malignancy, and have many non-immune side effects that hamper their tolerability. Specifically, CNIs are nephrotoxic, a side effect of significant concern in renal transplantation. Steroids exacerbate osteoporosis and hyperlipidemia, and cause avascular osteonecrosis. Both classes of agent worsen glucose tolerance and hypertension, and are associated with cosmetic effects causing non-compliance. As such, methods of transplantation that lessen the dependence on chronic immunosuppression stand to reduce the risk and expense of transplantation. They must, however, also prevent rejection. Development of alternate therapies that help to minimize the need for lifelong immunosuppression, or eliminate entirely the need for drugs through the induction of tolerance, are therefore of great interest.

Regulatory CD4+CD25+ T cells (Treg) derived from the thymus and/or peripheral tissues have been demonstrated to broadly control T cell reactivity (14). Importantly, Tregs have been shown to control immune responsiveness to alloantigens and significantly contribute to operational tolerance in transplantation models (15, 16). However, there have been limited efforts to harness the therapeutic potential of directly isolated CD4+CD25+ Treg cells for controlling graft rejection and inducing transplantation tolerance, such as for kidney transplants. In order for CD4+CD25+ Treg cells to be used as a clinical treatment, the following cell properties could be necessary: ex vivo generation of sufficient numbers of cells, migration in vivo to sites of antigenic reactivity, ability to suppress rejection in an alloantigen-specific manner, and survival/expansion after infusion for a critical, but currently unknown, period of time. Our published work and that of other investigators has demonstrated 1) the feasibility of expanding Treg ex vivo, 2) the ability of these cells to downregulate allogeneic immune responses in vitro, and 3) the efficacy of Treg for prevention of allograft rejection in animal models (15, 16). We have developed strategies for the ex vivo expansion of naturally occurring human Tregs (nTregs) that allow for the practical employment of this cellular therapy in the clinic. **Our central hypothesis is that sufficient human nTreg can be expanded ex vivo and used to both prevent renal transplant rejection and facilitate the reduction and subsequent withdrawal of drug-based immunosuppression**. This study will allow for us to define the safety of **Tr**eg **a**doptive **c**ellular **t**ransfer (**TRACT**) in living donor renal transplant recipients that draws upon our extensive preclinical experience with expanded Tregs, as well as our recognized clinical expertise with designing immunosuppressive regimens compatible with this type of therapeutic cell transfer.

1.1 Background

Significant progress has been achieved in the field of renal transplantation. One year graft survival approaches 89% for deceased donor kidney transplants and 94% for living related kidneys (http://www.unos.org). The use of combined immunosuppressive therapy is effective at controlling acute rejection with a calcineurin inhibitor, mycophenolate mofetil (MMF) and steroids or lymphodepletion induction followed by maintenance immunosuppression with a calcineurin inhibitor plus MMF (1-5). Graft survival depends on the chronic, daily use of these immunosuppressive agents. Although critical to graft survival, immunosuppressive agents have significant and potentially life- threatening toxicities. Because of their nonspecific mechanism of action, they broadly suppress immune function, rendering recipients susceptible to life-threatening infections and an increased risk of malignancies. Nephrotoxicity and hypertension are the principal dose-limiting side effects of the calcineurin inhibitors cyclosporine and tacrolimus. 80% of cardiac transplant recipients exhibit chronic renal compromise within 5 years due to the nephrotoxicity of the calcineurin inhibitors, and 29% of those with renal impairment progress to renal failure and require dialysis or renal transplantation. The cost of maintaining a transplant with immunosuppression is approximately $15,000 to $25,000 per year. Finally, even with perfect compliance, chronic rejection results in a fixed rate of graft loss over time. Retransplantation is expected to consume approximately one-third of renal allografts available for transplantation. If the need for these agents could be reduced or eliminated, it would be a significant milestone in transplantation. These unavoidable toxicities are what motivate individuals caring for transplant recipients to pursue the induction of tolerance (17).

1.2 Investigational Agent

Increasing evidence suggests that transplantation tolerance can be achieved through peripheral mechanisms, particularly immune suppression/regulation. Over the past decade, with the identification of a potent suppressor cell type, namely CD4+CD25+ “natural regulatory T cells” (nTreg), the importance of active immune regulation/suppression to the processes of graft rejection and tolerance has been greatly appreciated (18). Both nTreg and transplant-induced CD4+CD25+ Treg (iTreg) are expandable in the periphery by alloantigen and play important roles in controlling graft rejection and possibly lead to allograft tolerance. Numerous previously established tolerance protocols in rodents have focused upon generation and/or expansion of alloAg-specific CD4+CD25+ cells using therapeutic manipulations in the transplant recipient (19). Unfortunately, none of these approaches have been shown to expand CD4+CD25+ clones specifically and efficiently. One intriguing alternative for the clinical application of Tregs is to directly isolate and expand autologous CD4+CD25+ nTreg ex vivo, with re-introduction of these cells for the prevention of graft rejection and potentially induction of donor-specific tolerance – an approach termed **Treg adoptive cellular transfer (TRACT).**

Peripheral tolerance is mediated by multiple mechanisms such as anergy and/or suppression of effector T cell populations by regulatory T cells. Although different cell subsets with regulatory activity have been described, including CD4+CD25+Foxp3+, CD8+CD28-, and TCR+CD4- CD8-, studies have concentrated on the role of CD4+CD25+Foxp3+ T cells (19). Among the CD4+ T cell population, the

CD4+CD25+Foxp3+ regulatory T cell (Treg) have been demonstrated to suppress a variety of T cell dependent immune responses. Although studies have suggested that Treg downregulate both Th1 and Th2 mediated immune responses mainly through IL-10 and TGF-beta production), direct cell-cell contact has also been postulated as a mechanism of action. Importantly, Treg cells are inducible, can be antigen specific, and need to be activated through their TCR in order to wield their suppressive activities (18). Although Treg must encounter antigen to exert these effects, once activated they suppress in an antigen nonspecific manner, presumably through the release of immunosuppressive cytokines such as IL- 10 and TGF-beta.

It is now clear that Treg represent a critical pathway through which the immune system can actively control immune responses. Therefore, ex vivo induction of Treg might be extremely useful for therapeutic application in a variety of T cell mediated diseases. One difficulty in devising strategies for the clinical application of Tregs is their relative rarity. Tregs represent only 5-10% of the whole CD4+ T cell pool in mice and < 5% in humans (20). Isolation of a sufficient number of Tregs from a single donor for effective use would therefore be impossible. To circumvent this barrier, ex vivo expansion of Treg has been employed. Studies have shown that Treg can be effectively expanded in the presence of immobilized anti-CD3 mAb plus IL-2, or with antiCD3/CD28 coated microbeads and IL-2 (18). This approach allows for expansion of the entire population of Tregs, while maintaining a diversified repertoire, so-called polyclonal Tregs. Infusion of ex vivo expanded polyclonal Tregs has been shown to delay or even prevent GVHD in mice. Alternatively, Tregs can be expanded and, at the same time, selected for their capacity to recognize allogeneic antigen, so that antigen specific T reg can be obtained. Such antigen specific ex vivo expanded Treg have also been shown to delay or prevent GVHD in mice (21, 22), as well as to prevent organ transplant rejection (23).

Alloantigen-specific CD4+CD25+ cells are presumably generated in transplant recipients through multiple but not mutually exclusive pathways. First naturally selected thymic-origin T reg clones able to recognize alloantigens have been demonstrated in both naïve and transplanted hosts (i.e., natural CD4+CD25+). Second, alloantigen-specific Treg may be produced following transplantation through a process of positive selection in the thymus via alloantigen presentation. Third, a process of so-called "infectious tolerance" may be observed, where CD4+CD25+ Treg in the periphery convert CD4+CD25- cells into Treg through contact dependent and/or independent mechanisms. Both naturally and alloantigen-selected Treg are expandable in the periphery by alloantigens and play important roles in controlling graft rejection and possibly lead to allograft tolerance. Numerous previously established tolerance protocols in rodents have focused upon generation and/or expansion of alloantigen-specific CD4+CD25+ cells using therapeutic manipulations in the transplant recipient (15). Examples of such tolerizing strategies include using monoclonal antibodies (mAb) specific for CD3, CD4 or CD154, a combination of 1,25-dihydroxyvitamin D3 with mycophenolate mofetil, pre-transplant donor blood transfusion, and administration of tolerogenic or regulatory dendritic cells. Unfortunately, none of these approaches have been shown to expand CD4+CD25+ clones specifically and efficiently. Thus, therapeutic application of directly isolated natural CD4+CD25+ cells from naïve mice (and potentially human transplant recipients) is an intriguing approach for preventing renal transplant rejection and achieving operational tolerance. Indeed, several recent studies including our own have shown that fresh or ex vivo-activated human CD4+CD25+ Treg cells can prevent the development of GVHD and inhibit

skin graft rejection in murine models (15,16, 24). Importantly these studies have failed to observe undesirable pan-immunosuppression as a consequence of infusion with unprimed CD4+CD25+ Treg cells, which generally recognize a broad range of antigens and can suppress a variety of immune responses such as autoimmunity, tumor, infection and graft rejection. This latter observation raises the possibility that polyclonal Treg can acquire more restricted antigenic specificity following transfer to recipients.

1.3 Preclinical data in support of adoptive therapy with regulatory T cells (TRACT)

The term 'adoptive immunity' was first coined in 1954 by Billingham et al. (25), who were able to show that passive transfer of primed immune cells can generate immunity in the recipient.
Subsequently, numerous animal studies have demonstrated the effectiveness of this adoptive transfer of immunity towards cancer and infectious disease (26, 27).

Moreover, the use of IL-2 permitted, for the first time, the ex-vivo culture and expansion of T cells in humans (28). In addition, many transplant researchers found that CD4+ T cells were responsible for donor-specific tolerance, and it was the study by Hall et al. (29) which concluded that transplant tolerance was mediated by CD4+CD25+ cells. In this study they showed that in cyclosporine-treated rats with long-term cardiac allograft survival, the adoptive transfer of CD4+CD25+ T cells resulted in tolerance. The application of Tregs in the context of organ transplantation is supported further by the seminal work by Sakaguchi et al. (30), who showed that Tregs from naive mice prevented rejection of allogeneic skin grafts in T cell deficient nude mice given CD25– T cells. Subsequently, a series of preclinical rodent models of skin and cardiac transplantation demonstrated that Tregs present in the recipient at the time of transplantation are critical in the induction and maintenance of tolerance (reviewed in 14). In support of such studies, Lechler et al have also generated Treg lines in vitro, and shown that these Tregs are very effective at inducing survival of MHC-mismatched heart allografts (18). Furthermore, in a murine skin transplant model following thymectomy and partial T cell depletion, investigators have demonstrated the ability of in-vitro-expanded Tregs in inducing donor-specific transplantation tolerance (23). The importance of adoptive Treg therapy in transplantation is supported further in mouse models of bone marrow transplantation, where the transfer of freshly isolated Tregs together with the bone-marrow allograft has been shown to ameliorate GVHD and facilitate engraftment (21). GVHD was also the first model in which it was shown that the adoptive transfer of ex-vivo-expanded donor Tregs was highly effective in preventing acute or chronic GVHD (22). Moreover, the adoptive transfer of Tregs has been shown to prevent rejection of pancreatic islet (32) and other organ allografts (33, 34). The use of currently available humanized mouse models of GVHD and allotransplantation (35, 36) has reinforced further the importance of Tregs in these settings. These models are based on the reconstitution of immunodeficient mice with human immune cells. Sagoo et al have recently shown the efficacy of human Tregs in preventing alloimmune dermal tissue injury in a humanized mouse model of skin transplantation (37). Furthermore, Nadig et al. (38) developed a human vessel graft model to study the in-vivo function of Tregs. Their results showed convincingly that grafts from mice reconstituted with peripheral mononuclear cells (PBMCs) alone exhibited extensive vasculopathy, whereas the co-transfer of Tregs prevented this process. Such adoptive transfer experiments in rodents, therefore, support the notion that tolerance requires 'tipping the balance' between reactivity and regulation. Importantly, the in vivo efficacy of human Tregs in humanized mouse models has correlated extremely closely with the

ability of these cells to mediate in vitro suppression of T cell proliferation, supporting the use of such in vitro suppression assays as a means of gauging the potency of human Tregs for potential clinical application.

In summary, over the past several years laboratories have demonstrated that nTreg isolated and expanded in the laboratory (ex vivo-expanded CD4+CD25+ nTreg) and used for therapeutic cell transfer can inhibit skin and heart allograft rejection in mouse models. Importantly these studies have failed to observe undesirable pan-immunosuppression as a consequence of infusion with CD4+CD25+ nTreg, which generally recognize a broad range of Ag and can suppress a variety of immune responses such as autoimmunity, tumor, infection and graft rejection. Our work has allowed us to optimize expansion of nTregs while maintaining potent antigen-specific suppression of graft rejection. In addition, we have developed assays allowing for the immune monitoring of transplant recipients following the adoptive transfer of expanded autologous nTregs. Importantly, our research (and that of others) suggests that transient depletion of lymphocytes in the allograft recipient is crucial to the efficacy of expanded nTregs introduced for TRACT, as detailed below.

1.4 Clinical Data to Date

Beneficial effects of Treg infusions on allograft survival were first reported in bone marrow transplantation models in which donor Tregs reduced the incidence of GVHD. The first human trial using Treg cell therapy by Trzonkowski et al. (39) involved two patients. The first patient had chronic GVHD 2 years post-bone marrow transplantation. After receiving 0.1 x 10^6/kg purified (using flow cytometry) ex-vivo-expanded Tregs from the donor, the symptoms subsided and the patient was withdrawn successfully from immunosuppression without evidence of recurrence. The second patient had acute GVHD at 1 month post-transplantation, which was treated with several infusions of expanded donor Tregs. Despite initial and transitory improvement, the disease progressed and resulted ultimately in the patient’s death. This was the first report to show that adoptive transfer of Tregs is well tolerated and thus was a major breakthrough.

Results of a larger Phase I/II study were reported in which a total of 23 patients receiving umbilical cord blood (UCB) stem cell transplants were enrolled into a Treg escalation trial (40). CD4+CD25hi Tregs were isolated from a third party UCB graft and expanded by anti-CD3/CD28-coated beads and recombinant IL-2 over a period of 18 days. Patients received expanded Tregs at doses ranging from 1 x 10^5 to 30 x 10^5/kg. Of note, the targeted Treg dose was achieved only in 74% of cases. Compared with the 108 historical controls, there was a reduced incidence of grades II–IV acute GVHD (from 61 to 43%; P = 0.05), although the overall incidence of GVHD was not significantly different.

Treg related toxicities were reported as follows:

Regulatory T Cell Infusional Toxicities (40)

	Grade 1	Grade 2	Grade 3
Vomiting	4 (17.4%)	0	0
Hypertension	2 (8.7%)	1 (4.3%)	1 (4.3%)
Hypotension	1 (4.3%)	1 (4.3%)	0
Neurologic	1 (4.3%)	1 (4.3%)	0
Sinus bradycardia	1 (4.3%)	0	0
Sinus tachycardia	1 (4.3%)	0	0

Fever	0	1 (4.3%)	0
Headache	0	1 (4.3%)	0
Hypoxia	0	0	0
Dyspnea	0	0	0
Rash	0	0	0
Creatinine	0	0	0
Rigor/chills	0	0	0
Bleeding/hemorrhage	0	0	0
Allergic Reactions	0	0	0

In a third trial (Phase I/II), conducted by Di Ianni et al. (41), 28 patients were enrolled who underwent haematopoietic stem cell transplantation for haematological malignancies. Patients received donor Treg without ex-vivo expansion and donor conventional T cells (Tcons) without any other adjuvant immunosuppression. Different dose regimens were used, ranging from 5 x 10^5/kg Tcons with 2 x 10^6/kg Tregs to 2 x 10^6/kg Tcons with 4 x 10^6/kg Tregs. As two patients receiving the latter regimen developed acute GVHD, compared with none of the other patients, the authors concluded that a dose of 1 x 10^6/kg Tcons with 2 x 10^6/kg Tregs is safe. Moreover, patients receiving Tregs demonstrated accelerated immune reconstitution, reduced cytomegalovirus (CMV) reactivation and a lower incidence of tumor relapse and GVHD when compared to historical controls. However, only 13 of the 26 patients survived, but this may have been because of pre-existing fungal infections and the harsh conditioning regimens that were used. No Treg transfusional toxicities were noted. With the results from stem cell-treated patients showing that Treg therapy is well tolerated, we believe it is now time to initiate phase 1 trials in solid organ transplantation.

1.5 Clinically applicable strategy for ex vivo expansion of nTregs

The basic requirements to expand anergic fresh Foxp3+CD4+CD25+ nTreg cells include triggering of TCR (signal 1), activation of costimulation pathway(s) (signal 2), and the presence of IL-2. Thus far, allogeneic antigen-nonspecific expansion using anti-CD3/CD28 mAb-coated beads plus IL-2 with the addition of other cytokines (TGF-beta) or pharmacologics (Sirolimus, all-trans retinoic acid) that promote Treg development seems to be the most efficient method for ex vivo expansion of both mouse and human nTreg cells. These allogeneic antigen- nonspecifically expanded nTreg cells have been shown to suppress alloactivation in vitro as well as to prevent allograft rejection and graft-versus-host disease in vivo. It has been suggested by some that donor alloantigen-reactive Tregs are superior to polyclonal Tregs by offering targeted regulation of an immune response (20). Allo-specific Tregs are currently generated using allogeneic antigen presenting cells to directly activate alloreactive Treg populations. However, this approach fails to control immune responses that derive from the indirect presentation of alloantigen by recipient antigen presenting cells (18). In fact, polyclonal activation has the advantage of expanding the entire circulating Treg repertoire, with further selection and expansion of alloreactive clones then taking place after adoptive transfer. Our polyclonal activation methodology for the expansion of autologous nTregs to cell numbers we believe to be clinically efficacious is detailed below.

1.6 The importance of conventional T cell depletion to leverage the therapeutic efficacy of Tregs

Studies have demonstrated that a high ratio of Tregs to conventional CD4+ T cells (Tconv), such as 1:1 – 1:2, is needed to prevent transplant rejection (42, 43). A high prevalence of Tregs

is therefore needed to thwart rejection by establishing a dominant tolerogenic milieu through bystander suppression that is later maintained locally by infectious tolerance (44, 45). Considering the relatively small percentage of nTregs at normal steady state in peripheral blood of humans (< 5% of circulating CD4+ T cells), a drastic change of Tconv to Treg balance is needed, and we believe best achieved through the deletion of host donor-reactive T cells and the infusion of an appropriate number of expanded nTregs. A review of total numbers of Tconv cells and Tregs in humans, and the efficacy of clinically available strategies for T cell depletion, is essential to the design of a rational approach to the clinical application of Tregs. As detailed by Tang et al, lymphdepletion using T cell depleting monoclonal (Alemtuzumab) or polyclonal antibodies (Thymoglobulin) can reduce the CD4+ Tconv pool by 95-99% to approximately 4.6 x 10^9 cells (20). In our TRACT IND, we have demonstrated the ability to isolate and expand sufficient nTregs in our GMP facility from leukopheresis product to consistently achieve a desired Treg:Tconv ratio to support our TRACT clinical trial.

1.7 Experience with expansion of Human Tregs

For application in clinical practices, all procedures, which include human nTreg cell isolation and expansion, facility, equipment, reagents, and quality-control measures, will need to be conducted in compliance with good manufacturing practice requirements. Besides stringent regulatory, logistic, and financial challenges, some unique technical challenges must be overcome for the success of human Treg cell immunotherapy. First, purification of bona fide nTreg cells is problematic because of potential contamination by activated T effector cells if purification is simply based on the expression of CD25high. Recent studies have highlighted the use of a variety of cell surface markers, such as CD127, CD49d, CD45RA, and CD25, to purify nTreg cells. Second, expression of FOXP3 is not necessarily an indicator of suppressive capacity, as activated human T effector cells also express FOXP3, albeit transient and/or at a lower level, without suppressive function. Studies have highlighted the ability of culture conditions, especially addition of the mTOR inhibitor rapamycin, to promote the survival and expansion of Tregs and deter the survival and expansion of T effector cells.

We employ an approach for the isolation/enrichment of human nTregs using the CliniMACS™ (Miltenyi Biotec) immunomagnetic selection platform technology with clinical grade reagents for an initial negative selection of cells using CD8 and CD19 specific reagents, followed by the positive selection of CD25+ cells, with subsequent nonspecific expansion of cells. Expansion was conducted in the presence of rapamycin and TGF-beta in order to preserve Foxp3 expression and limit the outgrowth of effector CD4+ T cells, CD8+ and CD19+ cells. Expanded human Tregs using our methodology provides robust suppression in a donor specific fashion (>95% suppression of MLR at Treg:Teff ratio of 1:1).

1.8 Mathews Center for Cellular Therapy (MCCT) at Northwestern Memorial Hospital

cGTP / cGMP Facility Description

The MCCT is a fully operational current good tissue practices (cGTP) / current good manufacturing practices (cGMP)-compliant clinical facility dedicated to manufacturing support of human cellular and tissue-based products intended for human use. The CTPF is approximately 3,800 square feet, including a Quality Assurance/Quality Control (QA/QC) Laboratory, Receiving area, Control Room, Library and classified areas (ISO Class 5, ISO Class 7 and ISO Class 8). The ISO Class 7 areas consist of four (4) individual work suites, a

material staging area and a Clean Corridor. In addition, Work Suite 4 has an attached ISO Class 5 Cold Room. The ISO Class 7 Clean Corridor connects the work suites with the gowning, de-gowning and material staging areas. The ISO Class 8 areas consist of separate material airlocks (in & out) and gowning and de-gowning areas. The work suites are of suitable size for the intended MCCT manufacturing processes performed. Classified areas of the MCCT are cleanrooms, and are supplied with HEPA-filtered air. The areas are temperature, humidity and environmentally monitored.

Operational oversight for the facility is provided by the dedicated Northwestern Memorial Hospital facility management team and the established MCCT Advisory Board (consortium of end users). Facility operating funds are derived from clinical trial grants supported through investigator-initiated investigational new drug (IND) (or device, IDE) applications, sponsor-supported funding, as well as from clinical program revenues and NMH operational funds. Facility staffing consists of a medical director, laboratory director, laboratory manager, quality assurance officer, and a technical support staff of trained medical technologists.

1.9 Approach

We propose to use our knowledge gained from small animal and preclinical human studies to embark on an investigational pathway for clinical application of ex vivo expanded human nTregs as adoptive cellular therapy (TRACT). This is both extremely innovative and potentially very significant – TRACT with recipient derived Tregs could allow for significant reduction and perhaps elimination of immunosuppressive medications in organ transplantation. Although TRACT with ex-vivo manipulated Tregs have recently been initiated in the context of stem cell transplantation for the prevention of graft versus host disease, our clinical trial represents the first use of recipient-derived Tregs in an adult kidney transplant setting. In this proposal, we will conduct a phase 1 trial of TRACT in 12 recipients of HLA mismatched living donor renal transplants. In order to reach our target number of subjects that are needed to complete the study, we plan to recruit and consent up to 20 subjects, as we anticipate either screen failure or withdrawal from the study is a possibility prior to initiation of study involvement. Initiation of study involvement is defined as the administration of the Treg cellular product (study drug). As Northwestern University has the largest living donor transplant program in North America (~ 160 patients per year) we are confident in our ability to recruit and enroll the patients required for this study in a timely manner.

We believe our Phase 1 dose-escalation, safety trial of TRACT will allow us to draw upon unique strengths and opportunities for collaboration at Northwestern that will help move the evaluation of this cellular therapy forward. The cellular manufacturing facility at Northwestern Memorial Hospital has the required resources and expertise to generate GMP quality product in the form of expanded nTregs. Dr. Anton Skaro is an experienced kidney transplant surgeon in the Comprehensive Transplant Center at Northwestern University. Dr. Joseph Leventhal, a co-investigator and IND sponsor is the director of the Section of Cell Based Therapy of the Comprehensive Transplant Center at Northwestern University. He is PI on two ongoing trials of stem cell therapies in renal transplantation. His laboratory has published repeatedly on the use of Tregs for prevention of transplant rejection. Dr. Ann LeFever directs the MCCT and has extensive experience in generating clinical grade cellular products under an IND adhering to GTP and GMP regulatory requirements. Drs. Lorenzo Gallon and James Mathew have extensive experience with the immune monitoring assays to be conducted as part of our secondary objectives for our Phase 1 dose-escalation, safety trial of TRACT in kidney

transplant recipients.

2 STUDY DRUGS

2.1 Standard of Care Pharmacologic Agents used in Kidney Transplantation
The immunosuppressive agents to be utilized in this clinical trial are described below. In addition, we describe the TRACT cell product in detail.

Mycophenolic Acid (Myfortic®) - (Novartis Pharmaceuticals)
Myfortic®, is an uncompetitive and reversible inhibitor of inosine monophosphate dehydrogenase (IMPDH), and therefore inhibits the de novo pathway of guanosine nucleotide synthesis without incorporation to DNA. Because T- and B-lymphocytes are critically dependent for their proliferation on de novo synthesis of purines, whereas other cell types can utilize salvage pathways, MPA has potent cytostatic effects on lymphocytes. Myfortic® delayed- release tablets are an enteric formulation of mycophenolate sodium that delivers the active moiety mycophenolic acid (MPA). Myfortic is an immunosuppressive agent. As the sodium salt, MPA is chemically designated as (E)-6-(4-hydroxy-6-methoxy-7-methyl-3-oxo-1,3- dihydroisobenzofuran-5-yl)-4-methylhex-4-enoic acid sodium salt. Its empirical formula is C17H19O6Na. The molecular weight is 342.32.

Myfortic® for this clinical trial will be available via prescription as capsules. It will be dosed twice daily by mouth. Myfortic is available for oral use as delayed-release tablets containing either 180 mg or 360 mg of mycophenolic acid. Inactive ingredients include colloidal silicon dioxide, crospovidone, lactose anhydrous, magnesium stearate, povidone (K-30), and starch. The enteric coating of the tablet consists of hypromellose phthalate, titanium dioxide, iron oxide yellow, and indigotine (180 mg) or iron oxide red (360 mg).

Adverse events reported in clinical trials of Myfortic® include, but are not limited to; neutropenia, gastrointestinal upset, diarrhea, agitation, anxiety, arthralgia, acne, alopecia, hirsutism, pruritus, epistaxis, rhinitis, sinusitis, angina pectoris, urinary frequency, weight gain or weight loss.

Alemtuzumab (Campath-1H, Mabcampath) - (Genzyme Pharmaceuticals)
CAMPATH®, is a recombinant DNA-derived humanized monoclonal antibody (Campath-1H) that is directed against the 21-28 kD cell surface glycoprotein, CD52. CD52 is expressed on the surface of normal and malignant B and T lymphocytes, NK cell, monocytes, macrophages and tissues of the male reproductive system. The Campath-1H antibody has an approximate molecular weight of 150kD.

In this clinical trial, alemtuzumab will serve as the induction agent and is the standard of care in renal transplantation at Northwestern University/Northwestern Memorial Hospital for more than a decade. Subjects receiving alemtuzumab will be premedicated with 50mg diphenhydramine hydrochloride, and 650mg of acetaminophen 30-60 minutes prior to the first CAMPATH-1H infusion. The dose of 30 mg will be diluted in 100cc sterile 0.9% NS and infused over 2 hours. The infusion line must contain an in-line 0.22-micron filter. Campath will be administered on the day of transplant (intraoperatively), and on post-operative day #2. Both

doses will be administered while the subject is in the hospital. Alemtuzumab is supplied in single-use clear glass ampoules containing 30 mg of alemtuzumab in 3 mL of solution. Because this drug is being dosed while the subject is in the hospital, the hospital pharmacy will be responsible for mixing the medication appropriately. Alemtuzumab (before it is diluted into the saline infusion bag) should be stored at 2 to 8 °C (36-46 °F). Once in solution it is to be used within 8 hours and may be stored at room temperature (15-30 °C) or refrigerated until infused. It is to be discarded and not used should it become frozen and it is to be protected from direct sunlight.

Adverse events reported in clinical trials include but, are not limited to; rigors, drug-related pyrexia, nausea, vomiting, hypotension, neutropenia, thrombocytopenia, anemia, pancytopenia, rash, fatigue, urticaria, dyspnea, headache and diarrhea.

Initial doses of alemtuzumab of greater than 30 mg are not well-tolerated. A patient who received 80 mg as an initial dose by IV infusion experienced acute bronchospasm, cough, and shortness of breath, followed by anuria and death. A review of the case suggests that tumor lysis syndrome may have played a role. There is no known specific antidote for alemtuzumab over-dosage.

Tacrolimus (FK506) - (Astellas Pharmaceuticals)
Prograf® is a macrolide immunosuppressant produced by *Streptomyces tsukobaensis.* Chemically, tacrolimus is designated as:
[3S-[3R*[E(1S*,3S*,4S*)],4S*,5R*,8S*,9E,12R*,14R*,15S*,16R*,18S*,19S*,26aR*]]-5,6,8,11,12,13,14,15,16,17,18,19,24,25,26,26a-hexadecahydro-5,19-dihydroxy-3-[2-(4-hydroxy- 3-methoxycyclohexyl)-1-methylethenyl]-14,16-dimethoxy-4,10,12,18-tetramethyl-8-(2- propenyl)-15,19-epoxy-3H-pyrido [2,1-*c*][1,4] oxaazacyclotricosine-1,7,20,21(4H,23H)-tetrone, monohydrate. The empirical formula of tacrolimus is $C_{44}H_{69}NO_{12}{\bullet}H_2O$ and it has a formula weight of 822.05.

In this clinical trial, tacrolimus will be available by prescription and is a standard of care medication. It will be dosed orally at 2.0 mg twice daily and doses will be adjusted by serum levels. Tacrolimus is available as 0.5 mg (oblong, light yellow), 1.0 mg (oblong, white) and 5 mg (oblong, grayish-red) capsules. Regardless of strength, all capsules should be stored at 25 °C (77 °F), with excursions permitted to 15 °C-30 °C (59 °F-86 °F). A liquid formulation is also available.

Adverse events reported in clinical trials include, but are not limited to; tremor, seizure, renal insufficiency, nausea, vomiting, hypertension, thrombocytopenic purpura, pruritus, rash, dyspepsia, paresthesia, insomnia, alopecia, and headache.

Sirolimus (rapamycin) - (Pfizer Pharmaceuticals)
Rapamune® is an immunosuppressive agent which inhibits T lymphocyte activation and proliferation that occurs in response to antigenic and cytokine stimulation by a mechanism that is distinct from that of other immunosuppressants. Sirolimus is a macrocyclic lactone produced by *Streptomyces hydroscopicus.* The chemical name of Sirolimus is;
(3S,6R,7E,9R,10R,12R,14S,15E,17E,19E,21S,23S,26R,27R,34aS)-9,10,12,13,14,21,22,23,24,25,26,27,32,33,34, 34a-hexadecahydro-9,27-dihydroxy-3-[(1R)-2-

[(1S,3R,4R)-4-hydroxy-3-methoxycyclohexyl]-1-methyl-ethyl]-10,21-dimethoxy-6,8,12,14,20,26-hexamethyl-23,27-epoxy-3H-pyridol[2,1-c][1,4]oxaazacyclohentriacontine- 1,5,11,28,29 (4H,6H,31H)-pentone. Its molecular formula is $C_{51}H_{79}NO_{13}$ and its molecular weight is 914.2.

Sirolimus is available by prescription and will be obtained that way from the subject's pharmacy of choice as it is a standard of care medication in this patient population. In this study, Sirolimus will be dosed orally at 2 mg a day and the dosage will be adjusted by serum levels. Sirolimus 1 mg (triangular-shaped, white) and 2 mg (triangular-shaped, yellow to beige) tablets are available in bottles of 100 tablets or in a Redipak® cartons of 100 tablets (10 cards of 10 tablets each). Sirolimus is also available in liquid form. Tablets should be stored at 20-25 °C (68 to 77 °F) and be protected from light.

Adverse events observed in clinical trials include but, are not limited to; hypercholesterolemia, hyperlipidemia, hypertension, rash, acne, arthralgia, diarrhea, hypokalemia, thrombocytopenia, fever, diarrhea and gastrointestinal upset.

Reports of overdose with Sirolimus have been reported, however, experience has been limited. In general, the adverse events listed above are consistent with those observed in overdose. General supportive measures should be followed in all cases.

2.2 Study Drug

TRACT Cellular Product
The source of Treg for this trial is the kidney transplant recipient. The cells will be obtained by leukopheresis not less than one week prior to or more than one year before the kidney transplant. The leukopheresis product will be cryopreserved until used for Treg cellular expansion.

Central Line Placement for Leukopheresis
Patients who do not possess a vascular access (dialysis catheter, AV fistula/graft) and/or are not yet on any form of dialysis will need to undergo the placement of a temporary catheter by Interventional Radiology at NMH to facilitate leukopheresis to obtain lymphocytes for the isolation and expansion of autologous Tregs. The placement of an external central line catheter device is a routine procedure which may be done under local or general anesthesia. Potential complications include bleeding, pneumothorax, hemothorax, or arrhythmia. Like all artificial devices, lines may become infected and require treatment with antibiotics and/or removal. The central line will be in place for an approximate period of 1-2 days.

Leukopheresis
This procedure requires 4-6 hours and will be performed through a pheresis catheter or the subject's hemodialysis access. The total volume outside the body at any time does not exceed 450 ml. The most common complication is hypocalcemia arising from citrate anticoagulation, and is usually mild or rarely severe with nausea, vomiting or arrhythmias. Symptoms are avoided with replacement solutions added during apheresis, slowing the flow rate, and/or supplemental oral antacids containing calcium. Other complications are infrequent, but include hypotension, vasovagal syncope and infection.

TRACT Cellular Product cGMP Manufacture

All cellular processing and manufacturing will be conducted in the cGTP/cGMP compliant MCCT facility at Northwestern Memorial Hospital. Our preclinical validation of our cGMP manufacturing process has demonstrated our ability to obtain the required doses proposed for this clinical trial and that these processes provide products meeting all required release criteria.

After obtaining informed consent from the subjects, mononuclear cells will be harvested by apheresis as described above. The cell pheresis product is cryopreserved using autologous serum and DMSO and stored in vapor phase liquid nitrogen until approximately 21 days prior to the desired cell administration.

CD4+CD25+ regulatory T cells are isolated using a large scale (CliniMACS ™ Instrument, Miltenyi Biotec) closed-separation system. This process uses a two-step column procedure that initially eliminates CD8+, CD19+ cells and then selects for CD25+ cells. The resulting population is > 70% CD4+CD25+ cells. These cells are then cultured in TexMACS™ medium (Miltenyi Biotec) containing human AB serum, IL-2, rapamycin, and TGF-beta. Treg stimulation is provided by the addition of CD3+/CD28+ coated beads (Miltenyi Biotec) at a 4:1 bead to cell ratio. The Treg cultures are expanded in Wilson-Wolf G-Rex 100 culture flasks.

Cultures are restimulated on Day 7 of culture, fresh IL-2 is added every other day and additional medium added throughout culture. The stimulatory CD3+/CD28+ beads are removed from the final product prior to infusion. In-process and final quality control evaluation is performed. The release criteria for the cellular product is: >70% viable; gram stain negative; endotoxin < 5 EU/kg; CD4+/CD25+ > 70%; CD8+.CD19+ <10%; aerobic, anaerobic and fungal sterility negative, mycoplasma negative; residual bead count <3000 beads per 10^8 cells; and able to elicit
>50% suppression at a 1:2 Treg:Teffector cell ratio in a mixed lymphocyte reaction.

TRACT infusion and related toxicities

TRACT cellular product that has met the release criteria as specified in the IND will be infused on day +60 post kidney transplant. Release of TRACT product for clinical use will be obtained from the GMP laboratory Director and the approval for infusion will be granted by the PI based upon the Certificate of Analysis (COA) of the cellular product.

The Tregs will be infused under the supervision of the PI for the study through a large bore (≥18G) peripheral IV at a flow rate of no greater than 10cc/minute. Hemodynamic and clinical monitoring of the research subject by study personnel will be carried out with infusion documentation every 15 minutes during the infusion and for an hour following the end of the infusion.

Subjects will be monitored every 15 minutes starting with the initiation of the Treg infusion and then at 15 minute intervals. Monitoring will be carried out by the PI/Research Study Nurse for an hour after the completion of the Treg infusion. Routine monitoring will be continued during the 4 hours post infusion by the Clinical Research Unit nurse assigned to the research subject (CRU). Dose limiting toxicities will be evaluated for at least 21 days post infusion.

The patient will be under careful monitoring during and after infusion of the nTreg to monitor for any changes which may indicate hypersensitivity or other infusion related toxicities. Potential toxicities related to the Treg infusion based upon published clinical experience (40) include vomiting, hypertension, hypotension, headache, fever, sinus bradycardia and sinus tachycardia.

Monitoring will include monitoring of respiration, changes in blood pressure, angioedema, etc. The infusion will be done in the Northwestern Clinical Research Unit, which is well stocked with supportive medication such as steroids and norepinephrine, if these are required on an emergent basis. Premedication with corticosteroids (100 mg hydrocortisone), diphenhydramine and acetaminophen is routinely performed as prophylaxis for adverse infusion-related side effects.

2.3 Dose Rationale

A key aspect of using Tregs for therapeutic cell transfer is achieving a marked reduction in the total pool of T cells in order to facilitate successful dynamic immune regulation by the limited number of infused Tregs. We have elected to use Alemtuzumab (Campath-1H) for robust lymphodepletion in this Phase 1 trial. The dosing strategy for Campath-1H proposed in this study is based upon our extensive single center experience in kidney/pancreas transplantation in well over 2000 patients, much of which has been published (46, 47). In addition, it draws upon the additional published experience of Campath-1H in renal transplantation (48). Importantly, a recently published randomized prospective clinical trial of Campath induction as compared to polyclonal Thymoglobulin (INTAC study, Hanaway M et al, N Engl J Med. 2011) demonstrated safety and efficacy of Campath induction in renal transplant recipients, with superior lymphodepletion using Campath as compared to Thymoglobulin. In our experience, Alemtuzumab facilitates early CNI conversion and steroid free maintenance immunosuppression with a low (< 10%) incidence of acute rejection. Dosing of tacrolimus, MMF, and Sirolimus in this study is based upon published experience with the use of these agents to achieve therapeutic effect/desired blood levels and represent standard of care approaches to maintain immunosuppression management.

The cell dosing strategy proposed for TRACT is based upon a desire to ascertain the safety of TRACT infusion using cell numbers that straddle the projected therapeutic window based upon the need for sufficient Tregs to interact with the overall conventional T cell population. As such this will provide the safety data for subsequent phase 2 trials which will examine efficacy of TRACT to provide donor specific immune regulation allowing for the safe withdrawal of drug based immunosuppression.

The cell dosing proposed in our IND is based upon our current understanding of how Tregs control immune responses – namely that the ratio of Tregs to conventional T cells is of tremendous importance. Studies have demonstrated that a high ratio of Tregs to conventional CD4+ T cells (Tconv), such as 1:1 – 1:2, is needed to prevent transplant rejection (42, 43). A high prevalence of Tregs is therefore needed to thwart rejection by establishing a dominant tolerogenic milieu through bystander suppression that is later maintained locally by infectious tolerance (44, 45). Considering the relatively small percentage of nTregs at normal steady state in peripheral blood of humans (< 5% of circulating CD4+ T cells), a change of Tconv to Treg balance is needed, and we believe best achieved through the deletion of host donor-reactive T cells using T cell depleting antibody (Alemtuzumab), followed by the infusion of an appropriate number of expanded nTregs. A review of total numbers of Tconv cells and Tregs in humans, and the efficacy of clinically available strategies for T cell depletion, is essential to the design of a rational approach to the clinical application of Tregs. As detailed by Tang et al, lymphodepletion using T cell depleting monoclonal (Alemtuzumab) or polyclonal antibodies (Thymoglobulin) can reduce the CD4+ Tconv pool by 95-99% to approximately 4.6 x 10^9 cells

(52). We have designed our Phase 1 trial to provide the delivery of Tregs in numbers that would span the expected residual number of Tconv cells following lymphodepletion as described with alemtuzumab, achieving a comparably high ratio of Treg to Tconv. In our TRACT IND, we have demonstrated the ability to isolate and expand sufficient nTregs in our GMP facility from leukopheresis product to consistently achieve a desired Treg:Tconv ratio to support our TRACT clinical trial.

The starting dose we have proposed of 0.5 x 10^9 cells would approximate 7 million cells/kg in a 70 kg adult recipient. This cell dose is comparable to the 20 million Treg/kg body weight cell dose used by Trzonkowska et al. in a recently reported trial of adoptively transferred Tregs to prevent remission in juvenile subjects with Type 1 diabetes (53). The Tregs used in this trial were sorted and expanded using CD3/CD28 beads in a comparable fashion to the cellular manufacturing process described in our IND. No SAEs were reported with the Treg infusions in the Trzonkowska trial. Two other published trials of adoptive therapy with Tregs can be used for further comparison. In the trial reported by Di Ianni et al (41), 4 million/kg body weight of freshly isolated Tregs were used in 5 subjects receiving an allogeneic stem cell transplant; no dose limiting toxicities related to the Treg infusion were described, despite the co-infusion of a comparable number of conventional T cells. Finally, in the trial reported by Brunstein et al (40), more than a dozen patients received selected/sorted, polyclonally expanded allogeneic Tregs at a dose of 6 million cells /kg body weight; no dose limiting toxicities were seen with these Treg infusions. The methodology used for expansion in this trial (CD3/CD28 beads, IL2, rapamycin) is very similar to that used in our manufacturing process. In summary, we believe the published clinical experience with Tregs for adoptive cell therapy support the safe use of a starting dose of 0.5x10^9 cells using the manufacturing strategy we have proposed in our IND.

3 Study Objectives

3.1 Primary Objective

Determine the safety of administering expanded Treg adoptive cell transfer (TRACT) in living donor renal transplant recipients. Additionally data will be obtained to see if TRACT leads to generation of donor specific hyporesponsiveness that may facilitate immunosuppression withdrawal and determine whether TRACT therapy leads to transplant rejection/allosensitization and/or nonspecific immunosuppression.

3.2 Primary Outcome Measures

Determine the safety profile when escalating doses of TRACT are administered to the patient population post-kidney transplant.

3.3 Secondary Objectives

The secondary objectives are to assess:

1. Incidence of adverse events developed during the duration of the study associated with renal transplantation and immunosuppression
2. Incidence, timing and severity of bacterial, viral and fungal infections
3. The absolute numbers of Treg after renal transplant in patients receiving TRACT compared to numbers of Treg prior to TRACT administration

4 Study Design

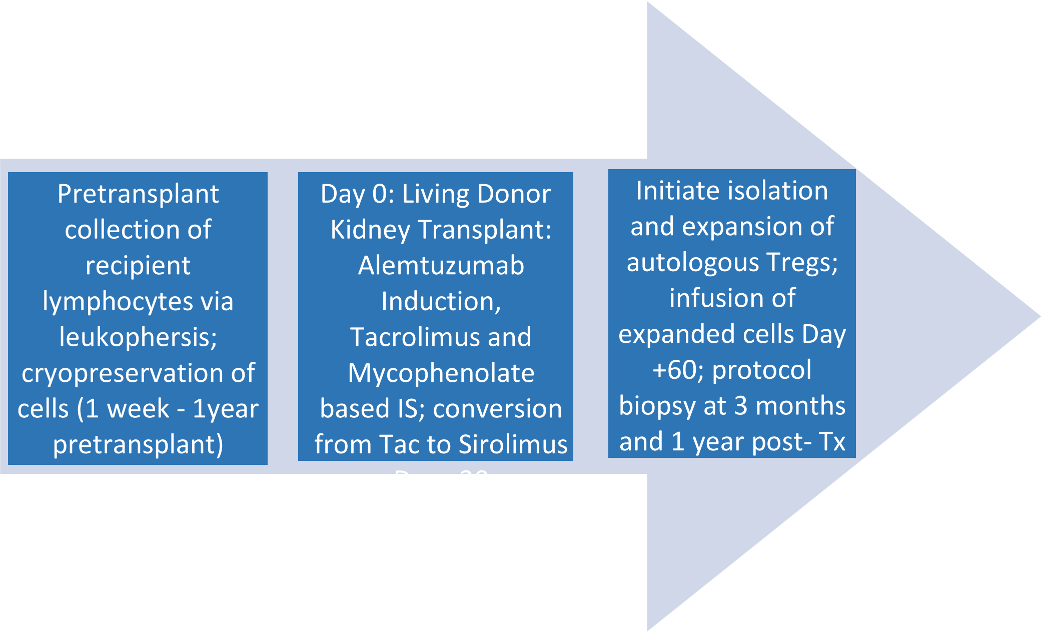

4.1 General Design

This is an open label, single-center phase I study of TRACT in living donor renal transplant recipients. This safety portion of this study will continue for six months but the patients will be followed for up to 60 weeks. Participants will receive "standard of care" immune suppression, living donor kidney transplantation and TRACT. Prior to the kidney transplant, blood will be collected for immunophenotypic analysis of circulating leukocytes by flow cytometry, as well as functional immune assays. **Dose escalation of nTregs will be based upon the absence of infusion related side effects and specific adverse events (subsequent rejection episodes, allosensitization, development of opportunistic infection) within 30 days of infusion.**

Patients will undergo living donor kidney transplantation using immunosuppression based upon the standard of care regimen using alemtuzumab and the avoidance of oral corticosteroids (46, 47). This immunosuppressive regimen has been used for more than a decade at Northwestern in more than 2000 kidney transplant recipients. All patients will receive two doses of alemtuzumab (Campath-1H, 30mg); one at the time of renal transplant, and one on post-operative day two to achieve peripheral T-cell depletion. Intravenous glucocorticoids will be administered prior to Campath administration to limit cytokine release syndrome in association with this monoclonal antibody, and continued for the first two days post-transplant. Thereafter, steroids will not be used for immunosuppression. All transplant recipients will be started on oral immunosuppressive therapy with MPA and tacrolimus prior to transplant. After 30 days, patients will be converted to the combination of Sirolimus and MPA, as the inhibition of IL2 production by CNIs would impair the survival of infused expanded Tregs.

An important consideration when using lymphocyte depleting induction agents such as alemtuzumab are the pharmacokinetics of the infused antibody. Premature introduction of Tregs in a recipient with circulating levels of alemtuzumab in a therapeutic range will lead to the unfortunate destruction of the infused cells. Published data on the half-life of alemtuzumab in patients without the cellular burden of hematologic malignancies is available from studies of Campath 1H in rheumatoid arthritis (49).Half-life of Campath in subjects receiving total treatment courses of 100mg to 400mg of Campath ranged from 5 to 9 days. Therefore, the infusion of TRACT in this study will be delayed until 60 days post kidney transplant to avoid Campath-related destruction of the infused cells.

TRACT infusion will be administered 60 days after kidney transplantation. Peripheral blood samples for immune monitoring studies (flow cytometry and immune function evaluation and urine samples for biomarker assessment will be collected pre-transplant and at 1, 3, 6, 9, and 12 months post-transplant as detailed below. Kidney biopsies will be obtained in accordance with standard of care clinical practice at 3 and 12 months post-transplant.

SCHEDULE OF EVENTS: SAMPLE COLLECTION FOR TRACT STUDY

Pre-Transplant

- Blood - no more than 100cc single blood draw, no more than 200cc in 30 day period:
- flow cytometry for lymphocyte subsets
- donor specific reactivity: MLR and Elispot
- recall antigen response - Candida, tetanus, CMV/EBV
- DSA
- Urine - 100cc for urinary m/microRNA assessment

1,3,6,9 and 12 months post -Tx

- Blood - no more than 100cc single blood draw, no more than 200cc in 30 day period:
- flow cytometry for lymphocyte subsets
- donor specific reactivity: MLR and Elispot
- recall antigen response - Candida, tetanus, CMV/EBV
- Urine - 100cc for urinary m/microiRNA assessment

Escalating doses of autologous expanded Tregs (TRACT) will be administered to the next cohort of patients if there are no observed serious adverse events within 30 days of TRACT infusion as follows:

Tier 1: 0.5 x 10^9 total expanded Tregs (n=3)
Tier 2: 1 x 10^9 total expanded Tregs (n=3)
Tier 3: 5 x 10^9 total expanded Tregs (n=3)
Tier 4: 8 x 10^9 total expanded Tregs (n=3)

Patients will be monitored at various time points, as described above for evidence of donor-specific transplantation tolerance using in vitro analyses, specifically mixed-leucocyte reaction assays (MLR). Graft function will be monitored clinically in the standard fashion. The overall goal for this phase I study is to demonstrate the safety of TRACT administration in kidney transplant recipients, and lay the foundation for a subsequent phase 2 trial. If we are successful, this approach could have for a transformative effect upon the field of solid organ and cellular transplantation.

The clinical site for recruitment, enrollment, and treatment of patients will be Northwestern Memorial Hospital. The Principal Investigator is part of a team with the most active living donor renal transplant program in the nation, with more than 160 such transplants performed in 2011. There already exists a robust infrastructure for combined therapeutic cell transfer (stem cell) and solid organ transplantation with two protocols for living donor renal transplants + HSC having been active at Northwestern for almost 5 years; Dr. Leventhal, the IND sponsor and co-investigator, serves as PI or co-PI on these trials (50, 51).

4.2 Primary Safety Endpoint

The primary safety endpoint is the evaluation of cellular related toxicities immediately and within 24 hours post-infusion of TRACT. Since TRACT is being administered to promote immunosuppression and prevent rejection, specific adverse events (subsequent rejection episodes, allosensitization, development of opportunistic infection) within 30 days of infusion will be monitored.

5 Subject Selection

The target population is adult recipients of immediately functioning living donor renal allografts. Immediate function will be defined as the absence of the need for hemodialysis in the first week following renal transplantation.

5.1 Inclusion Criteria

1. Patients who are males or females age 18-65 years.
2. Donor age 18-65 years.
3. No prior organ transplant.
4. Patients who are single-organ recipients (kidney only).
5. Women who are of childbearing potential must have a negative serum pregnancy test before transplantation and agree to use a medically acceptable method of contraception throughout the treatment period.
6. Subject (recipient) is able to understand the consent form and give written informed consent.

5.2 Exclusion Criteria

1. Known sensitivity or contraindication to Sirolimus, tacrolimus or MMF.
2. Patient with significant or active infection.
3. Patients with a positive flow cytometric crossmatch using donor lymphocytes and recipient serum.
4. Patients with PRA >20%.
5. Patients with current or historic donor specific antibodies.
6. Body Mass Index (BMI) of < 18 or > 35.
7. Patients who are pregnant or nursing mothers.
8. Patients whose life expectancy is severely limited by diseases other than renal disease.
9. Ongoing active substance abuse, drug or alcohol.
10. Major ongoing psychiatric illness or recent history of noncompliance.
11. Significant cardiovascular disease (e.g.):
    - Significant non-correctable coronary artery disease;
    - Ejection fraction below 30%;
    - History of recent myocardial infarction.
12. Malignancy within 3 years, excluding nonmelanoma skin cancers.
13. Serologic evidence of infection with HIV or HBV_sAg positive.
14. Patients with a screening/baseline total white blood cell count $\leq$4,000/mm^3; platelet count $\leq$100,000/mm^3; triglyceride $\geq$400 mg/dl; total cholesterol $\geq$300 mg/dl.
15. Investigational drug within 30 days prior to transplant surgery.

14. Anti-T cell therapy within 30 days prior to transplant surgery.

5.3 Subject Recruitment and Screening

The investigators will recruit patients in a manner that is sensitive to the inclusion of women and members of minority groups into this study. The renal failure population awaiting transplantation on the participating programs waiting lists is representative of the renal failure population in general. Subjects will be recruited from the patients who present to Northwestern University, Kovler Organ Transplantation Clinic, located in the Arkes Pavilion, 676 N. St. Clair Street, Suite 1900, Chicago, IL 60611.

5.3.1 Subject Information and Consent

Prior to any testing under this protocol, including screening tests and evaluations, written informed consent must be obtained from the subject in accordance with local practice and regulations. Whenever possible, the investigator will also be involved in this procedure. The background of the proposed study and the benefits and risks of the procedures and study will be explained to the subject. A copy of the informed consent document signed and dated by the subject must be given to the subject. Confirmation of a subject's informed consent must also be documented in the subject's medical records prior to any testing under this protocol, including screening tests and evaluations.

5.3.2 Avoidance of Coercion

It will be made clear to all patients that they may receive transplantation therapy (contingent on their medical suitability) without participation in this trial through participating or other UNOS approved clinical transplant centers. A list of UNOS approved centers will be made available to patients wishing this information.

5.4 Early Withdrawal of Subjects

5.4.1 When and How to Withdraw Subjects

Patients have the right to withdraw from the study at any time for any reason. The investigator has a right to withdraw patients from the study in the event of intercurrent illness, adverse events, protocol violations, or other reasons, which will be explained to the subject(s) should this occur.

Subjects must be withdrawn from the study for the following reasons:

-The subject desires to discontinue participation in this study.
-The subject is unwilling or unable to comply with the protocol.
-Subjects who prematurely withdraw from the study prior to the administration of TRACT will be replaced at the respective TRACT dose level. The reasons for withdrawal must be recorded in the subject's file.
-Subjects who withdraw from the study subsequent to the administration of TRACT will still be evaluated for safety and survival as part of their standard of care and will not be replaced at the respective TRACT dose level. The reasons for withdrawal must be recorded in the subject's file.

Patient withdrawal from the study for medical reasons or in association with an adverse event of

any kind will be followed for the life of the study as a study patient. Patients who voluntarily withdraw from the study will be counseled by the Principal Investigator as to the likely medical consequences of study withdrawal and will be referred for additional counseling (Pastoral Care, Social Work, Bioethics, and Psychiatry) if this is deemed appropriate. Upon withdrawal patients will be transitioned to standard immunosuppressive therapy and referred to a qualified transplant physician for follow-up.

5.4.2 Data Collection and Follow-up for Withdrawn Subjects

Even though subjects may be withdrawn prematurely from the study, it is imperative to collect at least safety and survival data on subjects who have received TRACT throughout the protocol defined follow-up period for that subject (though careful thought should be given to the full data set that should to be collected on such subjects to fully support the analysis). Such data is important to the integrity of the final study analysis since early withdrawal could be related to the safety profile of the study drug(s). If a subject withdraws consent to participate in the study, attempts should be made to obtain permission to record at least survival data up to the protocol-described end of subject follow-up period. It is a high priority to try to obtain at least safety and survival data on all subjects lost to follow-up and to note what methods were used to try to obtain this information from the subject (e.g. number of phone calls to subject, phone calls to next-of- kin if possible, certified letters, etc.). This is essential so one can state that the subject is truly lost to follow-up.

6 Study Related Therapies

6.1 Standard of Care Immunosuppression

Mycophenolate (MyF, Myfortic®)

The MPA/Myfortic will be initiated at 720-900 mg BID orally. The first dose will be given Day -2 pre-transplant. The agent will be given in an open label fashion. Once discharged from the hospital, the subject will obtain this medication via a prescription at a pharmacy of their choice.

Alemtuzumab (Campath-1H®)

The first dose will be 30mg given IV, and administered intra-operatively during renal transplantation. The subsequent dose will be 30mg given IV on postoperative day 2. The agent will be given in an open label fashion and will only be administered in the hospital.

Sirolimus (Rapamune®)

The Sirolimus will be given 2 mg daily starting at 30 days post-transplant to convert subjects off of tacrolimus. The dose will be modified to achieve 24 hour trough concentrations of 8-12 ng/ml by HPLC assay. The agent will be given in an open label fashion. Subjects will obtain Sirolimus via prescription at a pharmacy of their choice.

Tacrolimus (Prograf®)

Tacrolimus will be given 1-2 mg BID, beginning on Day -2. The dose will be modified to achieve 12 hour trough concentrations of 8-12 ng/ml. The agent will be given in an open label fashion. Tacrolimus will be discontinued at 30 days post-transplant and converted to Sirolimus therapy (see above).

Corticosteroids
Intravenous corticosteroids (prednisolone, Solumedrol®)

Time	Dose
Preop (Intraop)	500mg
POD#1	250mg
POD#2	125mg
POD#3	0mg

6.2 TRACT Cellular Product (Investigational Drug)

The source of Treg for this trial is the kidney transplant recipient. The cells will be obtained by leukopheresis not less than one week prior to or more than one year before the kidney transplant. The leukopheresis product will be cryopreserved until used for Treg cellular expansion.

6.2.1 Leukopheresis

Peripheral blood mononuclear cells (PBMCs) will be collected from eligible patients by apheresis at the Rube Walker Blood Center of Northwestern Memorial Hospital. Patients will undergo a single course of leukopheresis (25L collection) to collect approximately 4 x 10^9 PBMC. This procedure requires 4-6 hours and will be performed through a pheresis catheter or the subject's hemodialysis access. The total volume outside the body at any time does not exceed 450 ml. The most common complication is hypocalcemia arising from citrate anticoagulation, and is usually mild or rarely severe with nausea, vomiting or arrhythmias.
Symptoms are avoided with replacement solutions added during apheresis, slowing the flow rate, and/or supplemental oral antacids containing calcium. Other complications are infrequent, but include hypotension, vasovagal syncope and infection. Once collected, PBMCs will be immediately transported to the Northwestern Memorial Hospital GMP Cellular Manufacturing facility of the Mathews Center for Cellular Therapy (MCCT). The cell pheresis product is cryopreserved using autologous serum and DMSO and stored in vapor phase liquid nitrogen until approximately 21 days prior to the desired cell administration when Treg manufacturing is begun.

6.2.2 cGMP Manufacture

All cellular processing and manufacturing will be conducted in the cGTP/cGMP compliant MCCT facility at Northwestern Memorial Hospital. Our preclinical validation of our cGMP manufacturing process has demonstrated our ability to obtain the required doses proposed for this clinical trial and that these processes provide products meeting all required release criteria.

Isolation and separation of T-regulatory cell progenitors and mature T-regulatory cells will be performed using the Miltenyi CliniMACS™ System which is available for therapeutic applications. The CliniMACS™ System is based on ferromagnetic selection technology and Miltenyi has established a Master Drug File with Food and Drug Administration (FDA) for therapeutic applications. This clinical protocol, with a full description of the isolation procedures, will be submitted to the Northwestern University Institutional Review Board (IRB) for IRB approval prior to beginning any work. Miltenyi Biotec has provided a Letter of Cross Reference which is attached to the IND application for the investigation cellular product that

has been submitted to the FDA. The Miltenyi Letter of Cross Reference will permit the use of the device for manufacturing of the product once the FDA has approved the IND.

CD4+CD25+ regulatory T cells are isolated using a large scale (CliniMACS ™ Instrument, Miltenyi Biotec) closed-separation system. This process uses a two-step column procedure that initially eliminates CD8+, CD19+ cells and then selects for CD25+ cells. The resulting population is > 95% CD4+CD25+ cells. These cells are then cultured in TexMACS™ medium (Miltenyi Biotec) containing human AB serum, IL-2, rapamycin, and TGF-beta. Treg stimulation is provided by the addition of CD3+/CD28+ coated beads (Miltenyi Biotec) at a 4:1 bead to cell ratio. The Treg cultures are expanded in Wilson-Wolf G-Rex 100 culture flasks.
Cultures are restimulated on Day 7 of culture, fresh IL-2 is added every other day and additional medium added throughout culture. The stimulatory CD3+/CD28+ beads are removed from the final product prior to infusion. In-process and final quality control evaluation is performed. The release criteria for the cellular product is: >70% viable; gram stain negative; endotoxin < 5 EU/kg; CD4+/CD25+ > 70%; CD8+.CD19+ <10%; aerobic, anaerobic and fungal sterility negative, mycoplasma negative; residual bead count <3000 beads per 10^8 cells; and able to elicit
>50% suppression at a 1:2 Treg:Teffector cell ratio in a mixed lymphocyte reaction.

6.2.3 Expanded autologous TRACT Infusion

TRACT cellular product that has met the release criteria as specified in the IND will be infused on day 60 post kidney transplant. Release of TRACT product for clinical use will be obtained from the GMP laboratory Director and the approval for infusion will be granted by the PI based upon the Certificate of Analysis (COA) of the cellular product.

The T-Regs will be infused under the supervision of the PI for the study through a large bore ($\geq$18G) peripheral IV at a flow rate of no greater than 10cc/minute. Hemodynamic and clinical monitoring of the research subject by study personnel will be carried out with infusion documentation every 15 minutes during the infusion and for an hour following the end of the infusion.

Subjects will be monitored every 15 minutes starting with the initiation of the Treg infusion and then at 15 minute intervals. Monitoring will be carried out by the PI/Research Study Nurse for an hour after the completion of the Treg infusion. Routine monitoring will be continued during the 4 hours post infusion by the Clinical Research Unit nurse assigned to the research subject (CRU). Dose limiting toxicities will be evaluated for at least 21 days post infusion.

The patient will be under careful monitoring during and after infusion of the Treg to monitor for any changes which may indicate hypersensitivity or other infusion related toxicities. Monitoring will include monitoring of respiration, changes in blood pressure, angioedema, etc. The infusion will be done in the Northwestern Clinical Research unit, which is well stocked with supportive medication such as steroids and norepinephrine, if these are required on an emergent basis. Premedication with corticosteroids (100 mg hydrocortisone), diphenhydramine and acetaminophen is routinely performed as prophylaxis for adverse infusion-related side effects.

6.3 Treatment of Acute Rejection

Additional immunosuppressive medications will be given for treatment of acute renal allograft rejection. The diagnosis of rejection will be based on clinical symptoms and signs, laboratory tests, and confirmed by core renal allograft biopsy. The following therapy is suggested based upon biopsy results:
Mild (Banff I, IIA) - Methylprednisolone 0.5-1gram/day X 3 doses
Moderate/Severe (Banff IIB, III) - Anti-T cell agent.

6.4 Criteria for Conversion back to Tacrolimus

Patients who may be converted back to tacrolimus maintenance therapy include those experiencing a biopsy proven rejection episode and/or those patients who develop de novo DSA. In addition, at the discretion of the investigator, tacrolimus can be re-initiated in patients if the MPA or Sirolimus maintenance dose is significantly lowered (less than 360mg per day of MPA, 24 hour trough concentration less than 4 ng/ml for Sirolimus) for toxicity management. Tacrolimus dosing will be managed per center practice. As calcineurin inhibitors inhibit production of IL-2, an important cytokine for the survival of Tregs, reinitiation of tacrolimus may effect Treg function; restarting of tacrolimus would be noted in patient files and taken into consideration of data analysis.

6.5 Criteria for Addition of Oral Corticosteroids

Patients who may be started on prednisone maintenance therapy include those experiencing a biopsy proven rejection episode. In addition, at the discretion of the investigator, prednisone can be initiated if the MPA or rapamune maintenance dose is significantly lowered (less than 360 mg per day of MPA, 24 hour trough concentration less than 4 ng/ml for Sirolimus) for toxicity management. Prednisone dosing will be managed per center practice.

6.6 Preparation and Administration of Study Drugs

6.6.1 TRACT (Investigational Product)

The TRACT cellular product that has been released following confirmation of release criteria compliance will be infused as a fresh, non-cryopreserved product, intravenously in the Northwestern Clinical Research Unit by the PI or his designee. TRACT is given only once at the designated dose level per subject during this study.

6.6.2 Subject Compliance Monitoring

Study coordinators will track subject compliance using the solid organ transplant database (OTTR). This method of subject compliance monitoring is the standard of care in the Comprehensive Transplant Center for all patients.

6.6.3 Prior and Concomitant Therapy

1. **Antiviral prophylaxis**:
    - Oral valganciclovir for 3-6 months in the following CMV serostatus combinations: D+R-,
    - D-R+, D+R+: weekly monitoring with CMV quantitative PCR for 3 months and initiation of pre-emptive treatment with oral valganciclovir for evidence of CMV replication > 200 copies/ml
    - CMV serostatus combination D-R- no oral prophylaxis

2. **Antibacterial prophylaxis**
   - Cefazolin® 2gm q 8hr x 3 doses

3. **Anti-pneumocystis prophylaxis**
   - Trimethoprim/sulfamethoxazole single strength daily for 12 months. Patients with sulfa allergies receive oral atovaquone or alternative therapy.

4. **Antihypertensive** therapy per institution standards.

5. **Antihyperlipidemia** therapy per institution standards.

6. **Antiplatelet** therapy per institution standards.

7. **Anticoagulation** therapy per institution standards.

6.6.4 Packaging

All medications will come in standard packaging as per the pharmaceutical manufacturer and as supplied by their local pharmacy. There will be no special labeling since these are approved medications and will be obtained with a prescription after discharge. TRACT will be labeled as per FACT labeling requirements and as indicated in the investigational IND submission.

7 Study Procedures

All study procedures and assessments in this protocol are considered standard of care for living donor kidney transplantation, (those related to the investigational TRACT cellular product are specifically indicated as such). A clinical assessment will be completed at baseline. This assessment will include age, sex, race, weight, etiology of renal disease, method of dialysis prior to transplant, information on ABO and HLA matching of donor and recipient, results of current panel reactive antibody (PRA) and other pre-transplant crossmatches, CMV and EBV serologic profiles of donor and recipient, cold ischemia time and age of donor kidney, and a review of relevant past medical history. Beginning on the day of transplant surgery, patients will be monitored during their hospitalization and at regular scheduled intervals thereafter for the safety and efficacy described below.

7.1 Safety

Safety assessments for this study include:
Incidence of infectious episodes
Laboratory anomalies
Patient and graft survival

7.2 Infectious Episodes

Opportunistic infections, which occur during the course of the study, will be documented and include: CMV, Aspergillus, Candida, Pneumocystis, Cryptococcus, Listeria, Herpes Zoster, and Herpes Simplex. Other infections (bacterial) will also be noted.

7.3 Clinical and Laboratory Assessments

7.3.1 Pre-Transplant

The following may be performed during a clinic visit up to 3 months prior to transplantation:
Discussing with the potential subject potential inclusion into a study protocol Informed Consent discussed and signed (if the subject is ready to sign it)
A copy of the signed informed consent and HIPAA authorization is given to the subject.
The following will be performed for all patients within 48 hours before transplant:
Clinical assessment including history and physical examination Hematology: CBC with differential and platelet count
Blood chemistry: electrolytes (Na, K, Cl, Bicarb), BUN, Creatinine, glucose, calcium, AST, ALT, LDH, total bilirubin, total protein, albumin, uric acid, cholesterol (including LDL, and HDL), triglycerides
Coagulation parameters: PT, PTT Urinalysis,
urine culture (if possible) Pregnancy test:
Serum HCG
Tissue matching
T-cell and B-cell crossmatch
Donor and recipients CMV and EBV serology T/B cell
phenotyping
Elispot/ donor specific MLR
Mitogen Responsiveness In vitro assay

7.3.2 Post-Transplant

The following will be performed for all patients at the intervals described below:

Clinical assessment including history and physical examination: study days 7, 14, 28; study weeks 6, 8, 10, 12, 14, 16, 20, 24; study months 7, 8, 9, 10, 11, and 12
Hematology: study days 1, 7, 14, 28; study weeks 6, 8, 10, 12, 14, 16, 18, 20, 22, 24; study months 7, 8, 9, 10, 11, and 12
Blood chemistry: study days 1, 7, 14, 28; study weeks 6, 8, 10, 12, 14, 16, 18, 20, 22, 24; study months 7, 8, 9, 10, 11, and 12
24 hour creatinine clearance: 6 and 12 months post-transplant
T/B cell phenotyping: months 1, 3, 6, 9, 12,
Elispot/ donor specific MLR: months 1, 3, 6, 9, 12,
Recall/Mitogen Responses: months 1, 3, 6, 9, 12,
Protocol Renal Biopsy at 3 and 12 months

7.3.3 Core Renal Biopsies

All rejection episodes will be biopsy confirmed. In addition, protocol biopsies at three months and one year post-transplant will be performed.

7.4 Dose Limiting Toxicity (DLT) Determination in the Study

This is an open-label, single arm, dose escalation- de-escalation, phase I safety study which will examine the feasibility and safety of infusing autologous regulatory T cells (Tregs) into patients who have received a living donor kidney transplant. Tregs will be administered approximately 60 days post kidney transplantation.

According to the phase I dose escalation/de-escalation study design described below. The 4 dose levels are: 0.5 x 10^9, 1 x 10^9, 5 x 10^9, 8 x 10^9 Treg cells. All patients will be observed for a minimum of four hours following infusion and cell related toxicities monitored for the next three weeks. Patients with grade 3 or 4 toxicity during the infusion will have their cellular administration discontinued immediately and the event noted as a dose-limiting toxicity.

Adverse event information from the infusion will be used to determine whether the next subject within each cohort will receive the dose of cells associated with that dose level. The PI, IND Sponsor, IND Medical Monitor and study co-investigators will review toxicity reports and recommend whether or not it is safe to proceed at this dose level for the next patient. The next patient within a dose level will only be administered Treg after the first patient has been followed for at least 21 days and no evidence of dose limiting toxicity has been seen. The dose level for a given patient and the progression of doses from one cohort of patients to the next will be adjusted based on adverse events.

Adverse event information from the infusion will be used to determine whether or not dose escalation or de-escalation to the next level will occur. Adverse events will be graded using the Common Toxicity Criteria for Adverse Events, version 4.0e (NCI-CTCAE v4).

A dose-limiting toxicity (DLT) is defined as a clinically significant adverse event or abnormal laboratory value assessed as unrelated to the kidney transplant, intercurrent illness, or concomitant medications and occurring during the first 3 weeks after administration of the first dose that meets any of the following criteria: grade 3 or 4 non-hematologic toxicity (except for electrolyte disturbances responsive to correction measures within 48h, diarrhea, nausea and vomiting that responds to standard medical care, rash ≥ Grade 3 that response to supportive care, infusion relation reaction that are responsive to supportive care, infections and infectious complications related to patients neutropenia, elevated creatinine <3.5 X upper limit of normal, elevated AST< 8 X upper limit of normal, elevated ALT<8 X upper limit of normal, elevated bilirubin < 5X upper limit of normal, elevated PTT < 3.5 X upper limit of normal).

Dose escalation will only occur after patients, at a given dose level, have been followed for at least 21 days and no evidence of dose limiting toxicity has been seen. The dose level for a given patient and the progression of doses from one cohort of patients to the next will be adjusted based on adverse events. If a patient is discontinued due to concurrent illness unrelated to toxicity or organ rejection before they reach the end of the treatment cycle (21 days after the infusion), an additional patient will be enrolled at that same dose level.

**No. of Patients with DLT at a Given Dose Level**	**Escalation Decision Rule**
0 out of 3	Enter patient(s) at the next dose level.
≥ 2	Dose escalation will be stopped. This dose level will be declared the maximally administered dose (highest dose administered). Three additional patients will be entered at the next lowest dose level if only 3 patients were treated previously at that dose level.
1 out of 3	Enter at least 3 more patients at this dose level.<br>• If 0 of these 3 patients experience DLT, proceed to the next dose level.

	• If 1 or more of this group suffer DLT, then dose escalation is stopped and this dose is declared the maximally administered dose. Three additional patients will be entered at the next lowest dose level if only 3 patients were treated previously at that dose.
≤ 1 out of 6 at highest dose level below the maximally administered dose	This will be the Maximum Tolerated Dose (MTD). At least 6 patients will be entered at this dose.

Once a cohort is filled, enrollment into the next dose level will only begin after all patients in that cohort have passed through the DLT observation period. At that point, the PI, IND Sponsor, IND Medical Monitor and study co-investigators will review toxicity reports and recommend whether or not it is safe to proceed to the next cohort.

After escalation has stopped, de-escalation will begin at one dose level below the maximum achieved during escalation. The action taken depends on the number of patients previously treated at a dose level. If 3 or fewer patients have previously been treated, 3 patients are added; if 6 patients have previously been treated, this level will be declared to be the MTD. The MTD is defined to be the highest dose level at which no more than 1 of 6 treated patients experiences a DLT.

Patients within a cohort need not be accrued or treated at the same time; thus, accrual at a dose level should stop as soon as 2 patients at a dose level have experienced DLT at that level. There will be no intra-patient dose escalation or de-escalation.

7.5 Stopping Rules

The Principal Investigator may terminate the trial after consultation with the appropriate consulting staff at any time.

Patients with grade 3 or 4 toxicity during the infusion will have their cellular administration discontinued immediately and the event noted as a dose limiting toxicity.

The study as a whole will be halted if 2/5 patients develop grade 3-4 infusion reactions, or grade 4 organ toxicity within the first 30 days following Treg infusion. The study will also be halted if a death results from any of the criteria listed above.

If two of three consecutive subjects receiving experience severe steroid resistant acute rejection, vascular acute rejection, graft loss, malignancy, severe infection, and/or death in a period of 3 months, enrollment will be stopped and the study reassessed by the investigators, the IND sponsor and IND medical monitor.

7.6 Data Analysis

7.6.1 Analysis of the Primary Endpoints

Baseline descriptive statistics on all evaluable patients will be provided for demographic variables (age, sex, race/ethnicity), performance status, disease stage and status at the time of enrollment and treatment regimens previously used.

The NCI Common Terminology Criteria for Adverse Events, version 4 (CTCAE v4) will be used to evaluate toxicity. We will consider a toxicity to be an adverse event that is possibly, probably or definitely related to TRACT treatment. The maximum grade of toxicity for each category of interest will be recorded for each patient and the summary results will be tabulated by category and grade. We will describe all DLTs and other serious (≥ Grade 3) AEs on a patient-by-patient basis; descriptions will include dose level and any relevant baseline data. Data on the dose of cells they received will be tabulated.

7.6.2 Analysis of Secondary Endpoints

Clinical responses will be documented using the standard indicators of kidney function.

8 DATA SAFETY MONITORING PLAN

8.1 Monitoring and Quality Assurance

Investigator/Sub-investigators and clinical research staff meet monthly to review and discuss study data to include, but not limited to, the following:

- serious adverse events
- subject safety issues
- recruitment issues
- accrual
- protocol deviations
- breaches of confidentiality

All study participants on Phase I studies are also reviewed weekly for any toxicity or other untoward events. All toxicities encountered during the study will be evaluated on an ongoing basis according to the NCI Common Toxicity Criteria version 4.0 and recorded prior to each course of the investigational treatment. Any modifications necessary to ensure subject safety and decisions to continue or close the trial to accrual are also discussed during these meetings. All study data reviewed and discussed during these meetings will be kept confidential.

Any modifications necessary to ensure subject safety will be submitted to the IRB prior to implementation. The IRB will be notified of any change in the risk/benefit ratio which would affect whether the study should continue. All protocol deviations or breaches of confidentiality will be reported to the IRB according to IRB guidelines. If any literature becomes available which suggests that conducting this trial is no longer ethical the study will be terminated and the IRB will be notified of the new findings in the form of an Unanticipated Problem submission. All study treatment associated adverse events that are both serious and unexpected will be reported to the IRB according to the established guidelines.

All records related to this research study will be stored in a locked environment. Only the researchers listed on the first page of this form and their staff will have access to the research records. Any breach in subject confidentiality will be reported to the IRB in the form of an Unanticipated Problem submission.

9 Safety and Adverse Events

9.1 Definitions

Adverse Event

An ***adverse event*** (AE) is any symptom, sign, illness or experience that develops or worsens in severity during the course of the study. Intercurrent illnesses or injuries should be regarded as adverse events. Abnormal results of diagnostic procedures are considered to be adverse events if the abnormality:

- results in study withdrawal
- is associated with a serious adverse event
- is associated with clinical signs or symptoms
- leads to additional treatment or to further diagnostic tests
- is considered by the investigator to be of clinical significance

Serious Adverse Event

Adverse events are classified as serious or non-serious. A ***serious adverse event*** is any AE that is:

- fatal
- life-threatening
- requires or prolongs hospital stay
- results in persistent or significant disability or incapacity
- a congenital anomaly or birth defect
- an important medical event

Important medical events are those that may not be immediately life threatening, but are clearly of major clinical significance. They may jeopardize the subject, and may require intervention to prevent one of the other serious outcomes noted above. For example, drug overdose or abuse, a seizure that did not result in in-patient hospitalization, or intensive treatment of bronchospasm in an emergency department would typically be considered serious.

Unrelated ---Any reaction that is clearly due to extraneous causes AND that is likely to have been produced by the subject's clinical state or other modes of therapy administered to the subject.

Unlikely---Any reaction that does not follow a reasonable temporal sequence from the study intervention (in this case TRACT infusion) *OR* that is likely to have been produced by the subject's clinical state or other modes of therapy administered to the subject.

Possible---A reaction that follows a reasonable temporal sequence from the study intervention (in this case infusion of TRACT) *OR* that follows a known response pattern to the suspected drug *AND* that could not be reasonably explained by the known characteristics of the subject's clinical state or other modes of therapy administered to the subject.

Probable---A reaction that follows a reasonable temporal sequence from the study intervention (in this case TRACT infusion) *OR* that follows a known response pattern to the suspected drug *AND* that could not be reasonably explained by the known characteristics of the subject's

clinical state or other modes of therapy administered to the subject *AND* improves with the reduction in dose or cessation of the intervention.
Definite---A reaction that follows a reasonable temporal sequence from the study intervention (in this case TRACT infusion and toxicities occurring during the first 24 hours post-infusion) *AND* that follows a known response pattern to the suspected therapy

Severity of Adverse

Event Mild

Symptom(s) barely noticeable to subject or does not make subject uncomfortable; does not influence performance or functioning; prescription drug not ordinarily needed for relief of symptom(s) but may be given because of personality of subject.

Moderate

Symptom(s) of a sufficient severity to make subject uncomfortable; performance of daily activity is influenced; subject is able to continue in study; treatment for symptom(s) may be needed.

Severe

Symptom(s) cause severe discomfort; symptoms cause incapacitation or significant impact on subject's daily life; severity may cause cessation of treatment; treatment for symptom(s) may be given and/or subject hospitalized.

Rejection
See above under "**Treatment of Rejection"**

Life-threatening Infection or Malignancy
In the event that a recipient develops post-transplant lymphoproliferative disease, frank malignancy, or life-threatening infection, management will be based on standard medical therapy.

Pregnancy
Any pregnancy which occurs during this study will be reported as an SAE for tracking purposes. All pregnancies which are identified during this study will be followed to conclusion and outcome reported. All pregnancies will be reported on a SAE form.

Female patients will be instructed to immediately inform the Investigator of any pregnancies. There is currently no data relating to the effects of Treg on a developing fetus or nursing baby. The sponsor/investigator will counsel the patient, discuss the risks of continuing with the pregnancy and the possible effects on the fetus. Monitoring of the patient will continue until the conclusion of the pregnancy.

All adverse events that do not meet any of the criteria for serious should be regarded as ***non-***

serious adverse events.

Adverse Event Reporting Period

The study period during which adverse events must be reported is normally defined as the period from the initiation of any study procedures to the end of the study treatment follow- up. For this study, the study treatment follow-up is defined as 30 days following the last study related visit at month 12.

Preexisting Condition

A preexisting condition is one that is present at the start of the study. A preexisting condition should be recorded as an adverse event if the frequency, intensity, or the character of the condition worsens during the study period.

General Physical Examination Findings

At screening, any clinically significant abnormality should be recorded as a preexisting condition. At the end of the study, any new clinically significant findings/abnormalities that meet the definition of an adverse event must also be recorded and documented as an adverse event.

Post-study Adverse Event

All unresolved adverse events should be followed by the investigator until the events are resolved, the subject is lost to follow-up, or the adverse event is otherwise explained. At the last scheduled visit, the investigator-sponsor and/ or his study designee will instruct each subject to report any subsequent event(s) that the subject, or the subject's personal physician, believes might reasonably be related to participation in this study. The investigator-sponsor will notify the FDA, IRB and MCCT (cellular product manufacturer) of any death or adverse event occurring at any time after a subject has discontinued or terminated study participation that may reasonably be related to this study. The FDA, IRB and MCCT should also be notified if the investigator should become aware of the development of cancer or of a congenital anomaly in a subsequently conceived offspring of a subject that has participated in this study.

Abnormal Laboratory Values

A clinical laboratory abnormality should be documented as an adverse event if any one of the following conditions is met:

- The laboratory abnormality is not otherwise refuted by a repeat test to confirm the abnormality
- The abnormality suggests a disease and/or organ toxicity
- The abnormality is of a degree that requires active management; e.g. change of dose, discontinuation of the drug, more frequent follow-up assessments, further diagnostic investigation, etc.

Hospitalization, Prolonged Hospitalization or Surgery

Any adverse event that results in hospitalization or prolonged hospitalization should be documented and reported as a serious adverse event. Any condition responsible for surgery should be documented as an adverse event if the condition meets the criteria for an adverse event.

Neither the condition, hospitalization, prolonged hospitalization, nor surgery are reported as an adverse event in the following circumstances:

- Hospitalization or prolonged hospitalization for diagnostic or elective surgical procedures for a preexisting condition. Surgery should ***not*** be reported as an outcome of an adverse event if the purpose of the surgery was elective or diagnostic and the outcome was uneventful.
- Hospitalization or prolonged hospitalization required to allow efficacy measurement for the study.
- Hospitalization or prolonged hospitalization for therapy of the target disease of the study, unless it is a worsening or increase in frequency of hospital admissions as judged by the clinical investigator.

9.2 Recording of Adverse Events

At each contact with the subject, the investigator-sponsor and/or his designee must seek information on adverse events by specific questioning and, as appropriate, by examination. Information on all adverse events should be recorded immediately in the source document, and also in the appropriate adverse event module of the case report form (CRF). All clearly related signs, symptoms, and abnormal diagnostic procedures results should recorded in the source document and may be grouped under one diagnosis, if appropriate (ie., cough, runny nose, sore throat, fever [all at the same time] = upper respiratory infection).

All adverse events occurring during the study period must be recorded. The clinical course of each event should be followed until resolution, stabilization, or until it has been determined that the study treatment or participation is not the cause. Serious adverse events that are still ongoing at the end of the study period must be followed up to determine the final outcome. Any serious adverse event that occurs after the study period and is considered to be possibly related to the study treatment or study participation should be recorded and reported immediately.

9.3 Reporting of Serious Adverse Events

9.3.1 Notification by Investigator-Sponsor

A serious adverse event must be reported to the FDA, IRB and the MCCT within 24 hours of the investigator-sponsor's knowledge of the event. A Serious Adverse Event (SAE) form (FDA form 3500) must be completed by the investigator-sponsor and faxed to the FDA, IRB, and MCCT. The IRB specific serious adverse event form is also to be filled out and sent to the IRB. The investigator-sponsor will keep a copy of this SAE form on file at the study site in the regulatory binders specific for this study.

At the time of the initial report, the following information should be provided:

- Study identifier
- Study Center
- Subject number and/or initials
- A description of the event
- Date of onset
- Current status
- Whether study treatment was discontinued
- The reason why the event is classified as serious
- Investigator assessment of the association between the event and study treatment

The investigator must provide further information on the serious adverse event in the form of a

written narrative. This should include a copy of the completed Serious Adverse Event form, and any other diagnostic information that will assist the understanding of the event. Significant new information on ongoing serious adverse events should be provided promptly to the FDA, IRB and MCCT. Periodic reports of the SAE are made to the FDA, IRB and the cellular product manufacturer until the event is resolved and then a final report must be filled out and forwarded to both the FDA, IRB, and MCCT.

If a previous adverse event that was not initially deemed reportable is later found to fit the criteria for reporting, the study investigator-sponsor will submit the adverse event in a written report to the FDA and IRB as soon as possible, but no later than 15 calendar days from the time the determination is made.

9.4 Medical Monitoring

It is the responsibility of the Principal Investigator to oversee the safety of the study at his site. This safety monitoring will include careful assessment and appropriate reporting of adverse events as noted above, as well as the construction and implementation of a site data and safety-monitoring plan. Medical monitoring will include a regular assessment of the number and type of serious adverse events. Medical monitoring assessment and data will be reviewed prior to each dose escalation by the Principal Investigator, the IND sponsor and the IND medical monitor.

All adverse events will be reviewed weekly in a multidisciplinary format that will involve review by a transplant surgeon, a transplant physician, a pharmacist, and other members of the transplant staff. All Serious Adverse Events will be reviewed within Northwestern institutional guidelines and submitted to the appropriate regulatory bodies governing this study.

Renal failure has many known complications. Pre-existing conditions, which progress in keeping with known history, will not be expeditiously reported. This protocol is not designed to influence those pre-existing complications in any way. Therefore, only acute exacerbations that deviate from their natural history will be reported.

10 Data Handling and Record Keeping

10.1 Confidentiality

Information about study subjects will be kept confidential and managed according to the requirements of the Health Insurance Portability and Accountability Act of 1996 (HIPAA).

Those regulations require a signed subject authorization (separate from the informed consent document) informing the subject of the following:

- What protected health information (PHI) will be collected from subjects in this study
- Who will have access to that information and why
- Who will use or disclose that information
- The rights of a research subject to revoke their authorization for use of their PHI.

In the event that a subject revokes authorization to collect or use PHI, the investigator/sponsor, by regulation, retains the ability to use all information collected prior to the revocation of subject authorization. For subjects that have revoked authorization to collect or use PHI,

attempts should be made to obtain permission to collect at least vital status (i.e. that the subject is alive) at the end of their scheduled study period. Subjects are to notify the investigator/sponsor of their desire to withdraw their authorization in writing. A separate study specific HIPAA document will be given to the subject to read and sign prior to any study related procedures taking place.

10.2 Source Documents

Source data is all information, original records of clinical findings, observations, or other activities in a clinical trial necessary for the reconstruction and evaluation of the trial. Source data are contained in source documents. Examples of these original documents, and data records include: hospital records, clinical and office charts, laboratory notes, memoranda, subjects' diaries or evaluation checklists, pharmacy dispensing records, recorded data from automated instruments, copies or transcriptions certified after verification as being accurate and complete, microfiches, photographic negatives, microfilm or magnetic media, x-rays, subject files, and records kept at the pharmacy, at the laboratories, and at medico-technical departments involved in the clinical trial.

10.3 Case Report Forms

The study case report form (CRF) is the primary data collection instrument for the study. All data requested on the CRF must be recorded. All missing data must be explained (usually by a notation in the source documents). If a space on the CRF is left blank because the procedure was not done or the question was not asked, "N/D" should be written in. If the item is not applicable to the individual case, "N/A" should be written. All entries should be printed legibly in black ink. If any entry error has been made, a forensic correction (drawing a single straight line through the incorrect entry, then entering the correct data above it) should be made. All such changes must be initialed and dated. Errors are not to be erased or have "white out" placed over them.

10.4 Records Retention

It is the sponsor-investigator's responsibility to retain study essential documents for at least 2 years after the last approval of a marketing application in their country and until there are no pending or contemplated marketing applications in their country or at least 2 years have elapsed since the formal discontinuation of clinical development of the investigational product. These documents should be retained for a longer period off site (up to 10 additional years), should the need arise to refer back to the documents.

10.5 Auditing and Inspecting

The investigator/sponsor will permit study-related monitoring, audits, and inspections by the IRB, government regulatory bodies, of all study related documents (e.g. source documents, regulatory documents, data collection instruments, study data etc.).

Participation as an investigator in this study implies acceptance of potential inspection by government regulatory authorities and applicable University compliance and quality assurance offices.

11 Ethical Considerations

This study is to be conducted according to US and international standards of Good Clinical

Practice (GCP) (21 CFR312 and International Conference on Harmonization [ICH] guidelines), applicable government regulations and Institutional research policies and procedures.

This protocol and any amendments will be submitted to the Northwestern University Institutional Review Board (IRB), in agreement with local legal prescriptions, for formal approval of the study conduct. The decision of the IRB concerning the conduct of the study will be made in writing to the investigator/sponsor and a copy of this decision will be placed in the regulatory binder for the study. The investigator/sponsor will place a list of IRB members in the appropriate section of the regulatory binder.

All subjects for this study will be provided a consent form describing this study and providing sufficient information for subjects to make an informed decision about their participation in this study. This consent form will be submitted with the protocol for review and approval by the Northwestern University IRB. The formal consent of a subject, using the IRB-approved consent form, will be obtained before that subject is submitted to any screening or study procedure. This consent form must be signed by the subject and the investigator/sponsor-designated research professional obtaining the consent.

12 Study Finances

12.1 Funding Source

This study is being funded through an Intramural grant from Northwestern University. The Treg cellular product, for several patients is being supported by the Mathews Endowment for Regenerative Medicine through a grant to Joseph Leventhal (co-investigator). Subjects or their insurance carriers will not be charged for the manufacture of the investigational Treg cellular product.

12.2 Conflict of Interest

Any investigator who has a conflict of interest with this study (patent ownership, royalties, or financial gain greater than the minimum allowable by their institution, etc.) must have the conflict reviewed by the Office for the Protection of Research Subjects. All study personnel will have a conflict of interest form filled out, signed/dated and placed inside the regulatory binder for the study.

12.3 Subject Stipends or Payments

Subjects will receive free parking on the days of their study related visits. This is available only in Northwestern University "Lot C," located at 250 E. Huron Street which is attached to the clinic building (Galter Pavilion) and the hospital (Feinberg Pavilion) by covered pedestrian bridges over Huron Street. The subjects will receive a sticker which will placed on their parking card.

13 Publication Plan

13.1 Subject Data Protection

The subject will not be identified by name in any study reports, and these reports will be used for research purposes only. Various Government Health Agencies may inspect the records of this study. Every effort will be made to keep the subject's personal medical data confidential.

Data will be presented in abstract form for transplant conferences and also written up as a scientific paper at the end of the study when all data are analyzed. This paper will be presented for publication to a peer review journal. The principal investigator/sponsor hold the primary responsibility for publication.

Neither the complete nor any part of the results of the study carried out under this protocol, nor any of the information provided by the principal investigator/sponsor for the purposes of performing the study will be published or passed on to any third party without the consent of the principal investigator/sponsor.

14 References

1. Eggers PW: Effect of transplantation on the Medicare end-stage renal disease program. N. Engl. J Med. 1988; 318:223-229.
2. Hariharan S, Johnson CP, Bresnahan BA, Taranto SE, McIntosh MJ, Stablein D. Improved graft survival after renal transplantation in the United States, 1988 to 1996. N Engl J Med. 2000; 342:605-12.
3. Barry JM: Immunosuppressive drugs in renal transplantation. A review of the regimens. Drugs 1992; 44:554-566.
4. Suthanthiran M, Strom TB. Renal transplantation. N Engl J Med. 1994; 331:365-76.
5. Helderman JH, Van Buren DH, Amend WJ Jr, Pirsch JD. Chronic immunosuppression of the renal transplant patient. J Am Soc Nephrol. 1994; 4:S2-9.
6. Gaston RS. Maintenance immunosuppression in the renal transplant recipient: an overview. Am J Kidney Dis. 2001; 38:S25-35.
7. Pirsch JD, D'Alessandro AM, Sollinger HW, et al: Hyperlipidemia and transplantation: etiologic factors and therapy. J Am Soc Nephrol. 1992; 2:S238-S242.
8. Shaw LM, Kaplan B, Kaufman D: Toxic effects of immunosuppressive drugs: mechanisms and strategies for controlling them. Clin Chem. 1996; 42:1316-1321.
9. Boubenider S, Hiesse C, Goupy C, Kriaa F, Marchand S, Charpentier B: Incidence and consequences of post-transplantation lymphoproliferative disorders. J Nephrol. 1997; 10:136-145.
10. Fishman JA, Rubin RH. Infection in organ-transplant recipients. N Engl J Med. 1998; 338:1741-51.

11. DeMario MD, Liebowitz DN: Lymphomas in the immunocompromised patient. Semin Oncol. 1998; 25:492-502.

12. Sia IG, Paya CV: Infectious complications following renal transplantation. Surg Clin North Am. 1998; 78:95-112.

13. Pirsch JD, Miller J, Deierhoi MH, Vincenti F, Filo RS: A comparison of tacrolimus (FK506) and cyclosporine for immunosuppression after cadaveric renal transplantation. FK506 Kidney Transplant Study Group. Transplantation 1997; 63:977-983.

14. Wood KJ, Sakaguchi S. Regulatory T cells in transplantation tolerance. Nat Rev Immunol 2003; 3:199–210.

15. Xia G, He J, Zhang Z, Leventhal JR. Targeting acute allograft rejection by immunotherapy with ex vivo-expanded natural CD4+ CD25+ regulatory T cells. Transplantation 2006; 82:1749–55.

16. Xia G, He J, Leventhal JR. Ex vivo-expanded natural CD4+CD25+ regulatory T cells synergize with host T-cell depletion to promote long-term survival of allografts. Am J Transplant 2008; 8:298–306.

17. Newell KA, Larsen CP. Transplantation tolerance. Semin Nephrol 2007; 27:487–97.

18. Safinia N, Leech J, Hernandez-Fuentes M, Lechler R, Lombardi G. Promoting transplantation tolerance; adoptive regulatory T cell therapy Clin Exp Immunol. 2013 May; 172(2).

19. Xia G, Malathi S, Luo X. Prevention of allograft rejection by amplification of Foxp31CD4+CD25+ regulatory T cells. Translational Research 2009; 153:60–70.

20. Tang Q, Lee K. Regulatory T-cell therapy for transplantation: how many cells do we need? Curr Opin Organ Transplant 2012; 17:349–54.

21. Xia G, Kovochich M, Truitt RL, Johnson BD. Tracking ex vivo expanded CD4+CD2+1 and CD8+CD25+ regulatory T cells after infusion to prevent donor lymphocyte infusion-induced lethal acute graft-versus-host disease. Biol Blood Marrow Transplant 2004; 10:748–60.

22. Billingham RE, Brent L, Medawar PB. Quantitative studies on tissue transplantation immunity. II. The origin, strength and duration of actively and adoptively acquired immunity. Proc R Soc Lond B Biol Sci 1954; 143:58–80.

23. Dudley ME, Rosenberg SA. Adoptive-cell-transfer therapy for the treatment of patients with cancer. Nat Rev Cancer 2003; 3:666–75.

24. Porter DL, June CH. T-cell reconstitution and expansion after hematopoietic stem cell transplantation: ‘T’ it up! Bone Marrow Transplant 2005; 35:935–42.

25. Lotze MT, Line BR, Mathisen DJ, Rosenberg SA. The in vivo distribution of autologous human and murine lymphoid cells grown in T cell growth factor (TCGF): implications for the adoptive immunotherapy of tumors. J Immunol 1980; 125:1487–93.

26. Hall BM, Pearce NW, Gurley KE, Dorsch SE. Specific unresponsiveness in rats with prolonged cardiac allograft survival after treatment with cyclosporine. III. Further

characterization of the CD4+ suppressor cell and its mechanisms of action. J Exp Med. 1990; 171:141–57.

27. Sakaguchi S, Sakaguchi N, Asano M, Itoh M, Toda M. Immunologic self-tolerance maintained by activated T cells expressing IL-2 receptor alpha-chains (CD25). Breakdown of a single mechanism of self-tolerance causes various autoimmune diseases. J Immunol. 1995; 155:1151–64.
28. Tsang JY, Tanriver Y, Jiang S et al. Indefinite mouse heart allograft survival in recipient treated with CD4(+)CD25(+) regulatory T cells with indirect allospecificity and short term immunosuppression. Transpl Immunol 2009; 21:203–9.
29. Golshayan D, Jiang S, Tsang J, Garin MI, Mottet C, Lechler RI. In vitro-expanded donor alloantigen-specific CD4+CD25+ regulatory T cells promote experimental transplantation tolerance. Blood 2007; 109:827–35.
30. Joffre O, Gorsse N, Romagnoli P, Hudrisier D, van Meerwijk JP. Induction of antigen-specific tolerance to bone marrow allografts with CD4+CD25+ T lymphocytes. Blood 2004; 103:4216–21.
31. Cohen JL, Trenado A, Vasey D, Klatzmann D, Salomon BL. CD4(+)CD25(+) immunoregulatory T cells: new therapeutics for graft-versus-host disease. J Exp Med 2002; 196:401–6.
32. Sanchez-Fueyo A, Weber M, Domenig C, Strom TB, Zheng XX. Tracking the immunoregulatory mechanisms active during allograft tolerance. J Immunol 2002; 168:2274–81.
33. Joffre O, Santolaria T, Calise D et al. Prevention of acute and chronic allograft rejection with CD4+CD25+Foxp3+ regulatory T lymphocytes. Nat Med 2008; 14:88–92.
34. Raimondi G, Sumpter TL, Matta BM et al. Mammalian target of rapamycin inhibition and alloantigen-specific regulatory T cells synergize to promote long-term graft survival in immunocompetent recipients. J Immunol 2010; 184:624–36.
35. Shultz LD, Ishikawa F, Greiner DL. Humanized mice in translational biomedical research. Nat Rev Immunol 2007; 7:118–30.
36. Cao T, Soto A, Zhou W et al. Ex vivo expanded human CD4+CD25+Foxp3+ regulatory T cells prevent lethal xenogenic graft versus host disease (GVHD). Cell Immunol 2009; 258: 65–71.
37. Sagoo P, Ali N, Garg G, Nestle FO, Lechler RI, Lombardi G. Human regulatory T cells with alloantigen specificity are more potent inhibitors of alloimmune skin graft damage than polyclonal regulatory T cells. Sci Transl Med 2011; 3:83ra42.
38. Nadig SN, Wieckiewicz J, Wu DC et al. In vivo prevention of transplant arteriosclerosis by ex vivo-expanded human regulatory T cells. Nat Med 2010; 16:809–13.
39. Trzonkowski P, Bieniaszewska M, Juscinska J et al. First-in-man clinical results of the treatment of patients with graft versus host disease with human ex vivo expanded CD4+CD25+CD127– T regulatory cells. Clin Immunol 2009; 133:22–6.
40. Brunstein CG, Miller JS, Cao Q et al. Infusion of ex vivo expandedT regulatory cells in

adults transplanted with umbilical cord blood: safety profile and detection kinetics. Blood 2011; 117:1061–70.

41. Di Ianni M, Falzetti F, Carotti A et al. Tregs prevent GVHD and promote immune reconstitution in HLA-haploidentical transplantation. Blood 2011; 117:3921–8.
42. Hara M, Kingsley CI, Niimi M, et al. IL-10 is required for regulatory T cells to mediate tolerance to alloantigens in vivo. J Immunol 2001; 166:3789–3796.
43. Graca L, Thompson S, Lin CY, et al. Both CD4+CD25+ and CD4+CD25- regulatory cells mediate dominant transplantation tolerance. J Immunol 2002; 168:5558–5565.
44. Francis RS, Feng G, Tha-In T, et al. Induction of transplantation tolerance converts potential effector T cells into graft-protective regulatory T cells. Eur J Immunol 2011; 41:726–738.
45. Kendal AR, Chen Y, Regateiro FS, et al. Sustained suppression by Foxp3+ regulatory T cells is vital for infectious transplantation tolerance. J Exp Med 2011; 208:2043–2053.
46. Kaufman DB, Leventhal JR, Gallon LG, Parker MA. Alemtuzumab induction and prednisone-free maintenance immunotherapy in simultaneous pancreas-kidney transplantation comparison with rabbit antithymocyte globulin induction - long-term results. Am J Transplant. 2006 Feb; 6(2):331-9.
47. Walker JK, Scholz LM, Scheetz MH, Gallon LG, Kaufman DB, Rachwalski EJ, Abecassis MM, Leventhal JR. Leukopenia complicates cytomegalovirus prevention after renal transplantation with alemtuzumab induction. Transplantation. 2007 Apr 15; 83(7):874-82.
48. Hanaway MJ, Woodle ES, Mulgaonkar S, Peddi VR, Kaufman DB, First MR, Croy R, Holman J; INTAC Study Group. Alemtuzumab induction in renal transplantation N Engl J Med. 2011 May 19; 364(20):1909-19.
49. Isaacs JD, Manna VK, Rapson N, Bulpitt KJ, Hazleman BL, Matteson EL, St Clair EW, Schnitzer TJ, Johnston JM. CAMPATH-1H in rheumatoid arthritis--an intravenous dose- ranging study.Br J Rheumatol. 1996 Mar; 35(3):231-40.
50. Leventhal J, Abecassis M, Miller J, Gallon L, Ravindra K, Tollerud DJ, King B, Elliott MJ, Herzig G, Herzig R, Ildstad ST. Chimerism and tolerance without GVHD or engraftment syndrome in HLA-mismatched combined kidney and hematopoietic stem cell transplantation.Sci Transl Med. 2012 Mar 7; 4(124).
51. Leventhal JR, Mathew JM, Salomon DR, Kurian SM, Suthanthiran M, Tambur A, Friedewald J, Gallon L, Charette J, Levitsky J, Kanwar Y, Abecassis M, Miller J. Genomic Biomarkers Correlate with HLA-Identical Renal Transplant Tolerance. J Am Soc Nephrol. 2013 Sep; 24(9):1376-85.
52. Tang Q, Lee K. Regulatory T-cell therapy for transplantation: how many cells do we need? Curr Opin Organ Transplant 2012; 17:349–54
53. Trzonkowska NM et al. Administration of CD4+CD25highCD127- Regulatory T cells Preserves beta-cell function in Type 1 Diabetes in Children. Diabetes Care 2012 (35) 1817-1820.

15 Attachments

- New Project Submission Form
- HIPAA Response Form
- HIPAA Authorization Form
- Consent Form

- Study Procedures Flowchart/Table
- Investigator’s Brochure for Alemtuzumab
- Package Inserts for Mycophenalate Mofetil, Sirolimus, and Tacrolimus
